# Polycrystalline SnSe with a thermoelectric figure of merit greater than the single crystal

**DOI:** 10.1038/s41563-021-01064-6

**Published:** 2021-08-02

**Authors:** Chongjian Zhou, Yong Kyu Lee, Yuan Yu, Sejin Byun, Zhong-Zhen Luo, Hyungseok Lee, Bangzhi Ge, Yea-Lee Lee, Xinqi Chen, Ji Yeong Lee, Oana Cojocaru-Mirédin, Hyunju Chang, Jino Im, Sung-Pyo Cho, Matthias Wuttig, Vinayak P. Dravid, Mercouri G. Kanatzidis, In Chung

**Affiliations:** 1 School of Chemical and Biological Engineering, and Institute of Chemical Processes, Seoul National University, Seoul, Republic of Korea; 2 Institute of Physics (IA), RWTH Aachen University, Aachen, Germany; 3 Center for Correlated Electron Systems, Institute for Basic Science (IBS), Seoul, Republic of Korea; 4 Department of Chemistry, Northwestern University, Evanston, IL USA; 5 Chemical Data-Driven Research Center, Korea Research Institute of Chemical Technology, Daejeon, Republic of Korea; 6 Department of Mechanical Engineering, Northwestern University, Evanston, IL USA; 7 Advanced Analysis Center, Korea Institute of Science and Technology, Seoul, Republic of Korea; 8 National Center for Inter-University Research Facilities, Seoul National University, Seoul, Republic of Korea; 9 Department of Materials Science and Engineering, Northwestern University, Evanston, IL USA

**Keywords:** Solid-state chemistry, Thermoelectrics

## Abstract

Thermoelectric materials generate electric energy from waste heat, with conversion efficiency governed by the dimensionless figure of merit, ZT. Single-crystal tin selenide (SnSe) was discovered to exhibit a high ZT of roughly 2.2–2.6 at 913 K, but more practical and deployable polycrystal versions of the same compound suffer from much poorer overall ZT, thereby thwarting prospects for cost-effective lead-free thermoelectrics. The poor polycrystal bulk performance is attributed to traces of tin oxides covering the surface of SnSe powders, which increases thermal conductivity, reduces electrical conductivity and thereby reduces ZT. Here, we report that hole-doped SnSe polycrystalline samples with reagents carefully purified and tin oxides removed exhibit an ZT of roughly 3.1 at 783 K. Its lattice thermal conductivity is ultralow at roughly 0.07 W m^–1^ K^–1^ at 783 K, lower than the single crystals. The path to ultrahigh thermoelectric performance in polycrystalline samples is the proper removal of the deleterious thermally conductive oxides from the surface of SnSe grains. These results could open an era of high-performance practical thermoelectrics from this high-performance material.

## Abstract (web summary)

SnSe has a very high thermoelectric figure of merit ZT, but uncommonly polycrystalline samples have higher lattice thermal conductivity than single crystals. Here, by controlling Sn reagent purity and removing SnO_*x*_ impurities, a lower thermal conductivity is achieved, enabling ZT of 3.1 at 783 K.

## Main

More than 65% of the globally produced energy is lost as waste heat^1^. Thermoelectric power generators are semiconductor-based electronic devices that can turn such waste heat into electricity through the Seebeck effect^2^. This conversion process is free of motion or moving parts, thus can be an eco-friendly solution to recovering and using enormous amounts of waste heat to create electricity. The efficiency of thermoelectric semiconductors is assessed by the dimensionless figure of merit ZT = *S*^2^*σT*/*κ*_tot_ (refs. ^2–4^), where *S* is the Seebeck coefficient, *σ* is the electrical conductivity, *T* is the absolute temperature and *κ*_tot_ is the total thermal conductivity from the electrical (*κ*_ele_) and lattice vibration contribution (*κ*_lat_)^3^.

ZT values have been substantially improved by developing various strategies for increasing power factor (the product *S*^2^*σ*) or suppressing *κ*_lat_ in the past decade. They have been individually or multiply applied to representative thermoelectric systems such as lead chalcogenides^5^, skutterudites^6^ and half-Heusler compounds^7^. For example, an unusually high ZT roughly 2.2–2.5 around 920 K was achieved in PbTe–SrTe systems by applying multiple strategies of band engineering, endotaxial nanostructuring, hierarchical architecturing and non-equilibrium processing^8^. However, among the state-of-the-art thermoelectric systems, the most surprising and promising is the discovery of tin selenide (SnSe) as a top thermoelectric material^9–11^. This material combines two very desirable attributes: (1) highly effective inherent ultralow thermal conductivity and (2) very favourable electronic band structure with multiple bands contributing to the charge transport, thereby contributing to the ultrahigh power factor^9–11^. The innate strongly anisotropic and anharmonic crystal chemistry gives rise to intrinsically ultralow *κ*_lat_ of roughly 0.20 W m^–1^ K^–1^. As a result, its p-type pristine crystals exhibit a ZT of 2.6 at 913 K along the *b* axis^9^, and the Br-doped n-type crystals show a ZT of 2.8 at 773 K along the *a* axis^11^.

However, these extraordinarily high thermoelectric properties have been only observable in single-crystal SnSe samples while the polycrystalline versions show much poorer figure of merit^12–14^. In fact, many research groups have observed much higher thermal conductivity *κ*_lat_ values in polycrystalline SnSe samples than those reported for the single-crystal samples, despite the expected presence of additional phonon scattering mechanism from the grain boundaries (GBs)^15,16^. Accordingly, ZT values of the polycrystalline SnSe materials have been much lower than those of the single crystals. This has led to controversy regarding the ultralow *κ*_lat_ of SnSe as an intrinsic property and whether the exceptional ZT values of the single-crystal SnSe can ever be achieved in polycrystalline SnSe samples^15^. Indeed, given the high cost, lengthy and labour-intensive production, poor mechanical brittleness and high cleavability of the single-crystal SnSe samples, it is the polycrystalline samples that have a realistic chance to achieve mass production and commercial applications. Consequently, it has been a huge challenge to realize comparable or even higher thermoelectric performance in polycrystalline SnSe samples. Indeed, matching single-crystal thermoelectric performance in polycrystalline SnSe would be a major development; not only because of both maximum and average ZT during operating temperature range, but also due to the relative abundance of Sn and Se (in comparison to Te) as well as the lead-free nature of the compound.

Recently, we have revealed that this apparently higher *κ*_lat_ reported for polycrystalline SnSe samples is attributed to the presence of surface tin oxides (SnO_*x*_) on SnSe powders^12^. SnO_2_ has approximately 140 times higher *κ*_lat_ than SnSe^16^. When the SnSe powders covered with SnO_*x*_ thin films are compacted into dense pellets, high thermal conductivity SnO_*x*_ present at GBs provides natural percolation pathway for heat transport. In this case, the thermal conductivity is greatly enhanced, contrary to the general expectation that polycrystalline samples should have lower thermal conductivity than that of their single-crystal counterpart due to expected extensive GB phonon scattering. In fact, high thermal conductivity SnO_*x*_ phases can also easily grow on the surfaces of the single crystals, thereby often complicating the studies of the thermal transport properties. Further, the surface SnO_*x*_ can strongly scatter charge carriers, consequentially affecting both thermal and charge transport properties adversely, and as a result severely curtailing the promise of a cost-effective, eco-friendly, widely deployable thermoelectric material such as SnSe. Indeed, polycrystalline SnSe with minimal GB and surface phase of SnO_*x*_ would be a major advance in this context.

To initially mitigate this problem, we developed a postprocess of ball milling combined with chemical reduction for polycrystalline SnSe-based materials. This approach effectively removes SnO_*x*_ phase from surfaces and subsequent interfaces to reveal the exceptionally low *κ*_lat_ of roughly 0.11 W m^–1^ K^–1^ and near-single-crystal ZT of roughly 2.5 at 773 K (ref. ^12^). However, despite this great progress these samples still show higher *κ*_lat_ of roughly 0.84–0.32 W m^–1^ K^–1^ than the single-crystal SnSe with 0.47–0.24 W m^–1^ K^–1^ in the nearly entire temperature range 300–673 K suggesting the presence of persistent and pervasive presence of SnO_*x*_ in the samples. This continues to obscure the intrinsic thermal and charge-carrier transport properties of SnSe and, as a result, the true thermoelectric properties of SnSe have hitherto not yet been realized in polycrystalline samples of SnSe.

**Fig. 1:**
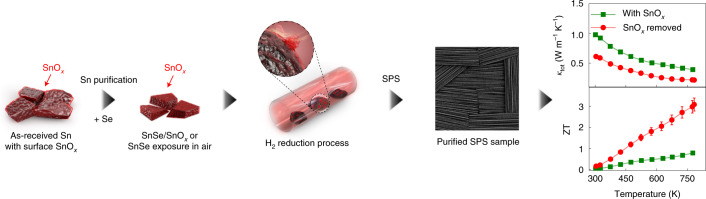
A schematic illustration of the process to remove surface tin oxides (SnO_*x*_) in polycrystalline SnSe, and to reveal the intrinsic thermoelectric properties of the material. Our facile two-step process involves the successive purification of the tin starting reagent and the synthesized SnSe samples. The use of the purified samples minimizes the presence of SnO_*x*_ in the SPS-processed dense pellets. As a result, the intrinsically ultralow thermal conductivity (*κ*_tot_) is finally uncovered in the purified sample (green squares on the right) in sharp contrast to the controversially high values in the untreated sample (red circles), leading to the record-high thermoelectric figure of merit, ZT, of roughly 3.1 among all bulk thermoelectric systems. Source data

Herein, we report that the tin (Sn) metal starting reagent, despite its 99.999% purity, is the culprit behind the formation of surface SnO_*x*_ in polycrystalline SnSe-based materials. To remedy this further, we have developed a facile and more efficient two-step process to remove the deleterious oxygen and minimize the presence of SnO_*x*_. Collectively, this further reduces the thermal conductivity and increases the power factor, thereby uncovering the extraordinarily high thermoelectric performance of polycrystalline SnSe, which reaches a ZT of roughly 3.1 at 783 K. A schematic illustration of this process is shown in Fig. 1.

## Purification process for SnSe

As-received elemental tin (Sn) reagent must be purified before use. Note that we use 99.999% purity Sn chunks, showing a characteristic silvery lustre. This was chemically reduced by a 4% H_2_/Ar flow for 6 h at 473 K, near the melting point of Sn, showing no visible change in surface colour and lustre afterwards. The metal was subsequently heated at 1,223 K in an evacuated ampule. This caused ash-like black residues to form at the top and entire surface of the resulting Sn ingot and it was unambiguously identified as SnO_*x*_ by far-infrared spectroscopy^17^ and atom probe tomography (APT) (Supplementary Figs. 1–3). After removing these residues, the melting-purification process was repeated until the ash-like black SnO_*x*_ residues were no more visible. The purified Sn reagent was confirmed to be nearly oxygen-free according to the APT analysis (Supplementary Fig. 3). We found that the purification of elemental selenium (Se) reagent had a negligible influence on thermoelectric properties of SnSe. After the purification of Sn, the synthesized SnSe samples were pulverized and further purified under a 4% H_2_/96% Ar flow at 613 K for 6 h. For the sake of the discussion, samples prepared by this two-step purification process are referred to as ‘purified’, while those not prepared by this process are denoted as ‘untreated’.

### Analysis of surface SnO_*x*_ in untreated and purified SnSe

The facile formation of surface SnO_*x*_ in polycrystalline SnSe samples is supported by our density functional theory (DFT) calculations (Supplementary Fig. 4 and Note). To probe the presence and distribution of surface SnO_*x*_ in both the untreated and purified SnSe samples, we first performed time-of-flight–secondary ion mass spectrometry (TOF–SIMS). This is a highly surface-sensitive technique providing chemical mapping at spatial resolutions down to a submicrometre scale, thereby providing the broad-range distribution of surface SnO_*x*_ at GBs. We mapped the SnOH^+^ species to reliably display the spatial distribution of tin-bound oxygen.

**Fig. 2:**
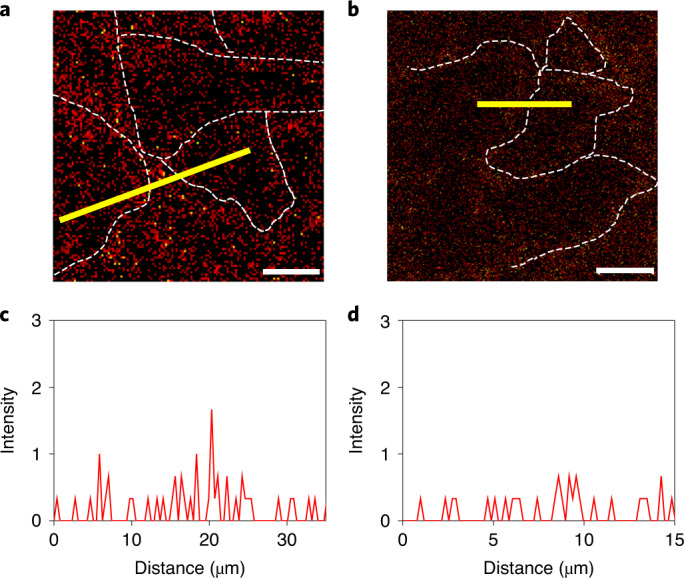
Distribution of SnO_*x*_ in untreated and purified polycrystalline SnSe samples obtained by TOF–SIMS. The surface of both SPS-processed specimens was sputtered to generate SnOH^+^ complex that is a relevant quantity to tin-bound oxygen. Accordingly, the SnOH^+^ map clearly represents the distribution of surface SnO_*x*_ on SnSe samples. **a**, The SnOH^+^ image for the untreated SnSe sample. **b**, The SnOH^+^ image for the purified SnSe sample. The red spots correspond to SnO_*x*_. The white dotted lines indicate GBs, which were defined with optical images taken on the corresponding regions. Scale bars are 10 μm. **c**, The concentration of SnO_*x*_ across the GB by a line profile (yellow solid line in **a**) for the untreated SnSe sample. **d**, The concentration of SnO_*x*_ across the GB by a line profile (yellow solid line in **b**) for the purified SnSe sample. The width of a line profile is 3 μm, in which the concentrations of SnO_*x*_ were averaged. The substantial decrease in surface SnO_*x*_ is clearly observed by our purification process. Source data

Figure 2a,b shows TOF–SIMS images of the untreated and purified spark plasma sintering (SPS) SnSe samples. Spread red spots correspond to the distribution of SnOH^+^, which are much fainter and less dense in the purified SnSe sample. The analysed data show that it has a factor of 7.4 lower SnO_*x*_ concentration than the untreated sample. After identifying the GBs in the corresponding optical images (Supplementary Fig. 5a,b), the line-profile scan for the SnO_*x*_ concentration was taken across them. It revealed that SnO_*x*_ is more abundant in the GBs than in the interior regions of SnSe crystallites (Fig. 2c,d). This is not surprising in view of the compaction process of SnSe powders, which are surface-covered with SnO_*x*_.

**Fig. 3:**
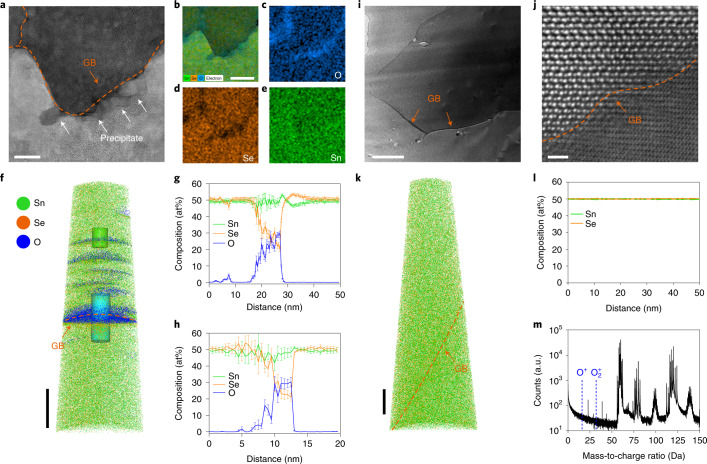
Distribution and composition of SnO_*x*_ in untreated and purified polycrystalline SnSe samples. **a**, HAADF–STEM image for the untreated polycrystalline SnSe sample, revealing SnO_*x*_ precipitates around the GBs as indicated by the white arrows. Scale bar, 20 nm. **b**, Elemental map recorded on the entire area of **a** by STEM–EDS. Scale bar, 50 nm. **c**–**e**, A joint image by overlaying the EDS signals directly arising from O (**c**), Se (**d**) and Sn (**e**) atoms, respectively. **f**, Three-dimensional APT reconstruction of the untreated polycrystalline SnSe specimen, presenting the spatial distribution of Sn (green), Se (orange) and O (blue) atoms. Scale bar, 50 nm. **g**,**h**, One-dimensional compositional profiles showing the content of Sn, Se and O atoms across the GB as enclosed by the blue cylinder (**g**) and across the oxygen-rich layer as marked by the green cylinder (**h**) in **f**, respectively. **i**, HAADF–STEM image for the purified SnSe sample, confirming the absence of SnO_*x*_ around the GBs. Scale bar, 200 nm. **j**, Magnified HAADF–STEM image focusing on the GB, showing two adjacent crystalline grains form the tightly jointed interface without intervening secondary phases. Scale bar, 1 nm. **k**, Three-dimensional APT reconstruction of the purified SnSe, representing the spatial distribution of Sn (green) and Se (orange) atoms. The O atoms are not detected, verifying the successful removal of SnO_*x*_ by our two-step purification process. Scale bar, 50 nm. **l**, One-dimensional compositional profile extracted across the GB, demonstrating an at% ratio of Sn and Se atoms that is nearly constant at unity over the specimen. **m**, The mass-to-charge ratio spectrum for the purified sample, confirming the absence of signals from O atoms as indicated by the blue dashed lines. The orange arrows and dashed lines in **a**, **f**, **i**, **j** and **k** indicate the GBs in the samples. Source data

We further investigated surface SnO_*x*_ in GB regions in the untreated SnSe sample using a spherical aberration-corrected scanning transmission electron microscope (STEM). A representative high-angle annular dark-field (HAADF)–STEM image shows the presence of abundant nanoscale precipitates, indicated by the white arrows, around the GB marked by the orange dashed line and arrow (Fig. 3a). The corresponding elemental map reveals that they are rich in oxygen and devoid of selenium with the negligible fluctuation in the tin concentration throughout the specimen, thereby being identified as SnO_*x*_ (Fig. 3b–e).

To spatially determine the distribution and composition of surface SnO_*x*_, we conducted APT analysis on the untreated SnSe sample. It quantitatively provides the three-dimensional distribution of constituent elements with equal sensitivity at a spatial resolution nearly down to the subatomic level, thereby serving as an effective tool to resolve secondary phases either in the matrix or trapped at GBs^18–20^. Figure 3f displays the three-dimensional reconstruction of the needle-shaped specimen from the untreated SnSe sample. The GB, marked by the orange arrow and dash line, is located by a much higher atomic counts due to the local magnification effect^20^. The high concentration O atoms are aggregated along the GB, coincident with our STEM observations. They also percolate into the grain forming SnO_*x*_ layers as observed in the upper area in Fig. 3f.

To quantitatively resolve their content with the greater statistical accuracy, one-dimensional compositional profiles were recorded at the oxygen-rich region, namely both across the GB as enclosed by the blue cylinder (Fig. 3g) and across the oxygen-rich layer as marked by the green cylinder (Fig. 3h) in Fig. 3f. In these regions, the O concentration exceeds roughly 15 at% with a maximum reaching roughly 30 at%, whereas the Se concentration drops by greater than 20%. Outside these, the former rapidly decreases and a compositional ratio of Sn to Se atom remains nearly constant at unity. The typical thickness of surface SnO_*x*_ layer at GBs is about 15 nm in the untreated SnSe sample according to both STEM and APT observations. Even nanoscale GB phases could considerably affect charge^21^ and thermal^22^ transport properties of materials, consequently inhibiting the observation of intrinsic values^21,22^.

A typical HAADF–STEM image for the purified SnSe sample does not show the presence of SnO_*x*_ at the GBs (Fig. 3i). The magnified image focusing on the GB shows that two adjacent crystalline grains form the tightly jointed interface without intervening secondary phases (Fig. 3j). The three-dimensional APT reconstruction (Fig. 3k) and one-dimensional compositional profile extracted across the GB (Fig. 3l) show that the distribution of Sn and Se atoms is nearly homogeneous at the same level over the specimen with a negligible discontinuity across the GB. No signal for the presence of O atoms is detected in the mass-to-charge ratio spectrum (Fig. 3m). The results confirm that our purification process effectively removes surface SnO_*x*_ from SnSe-based materials.

**Fig. 4:**
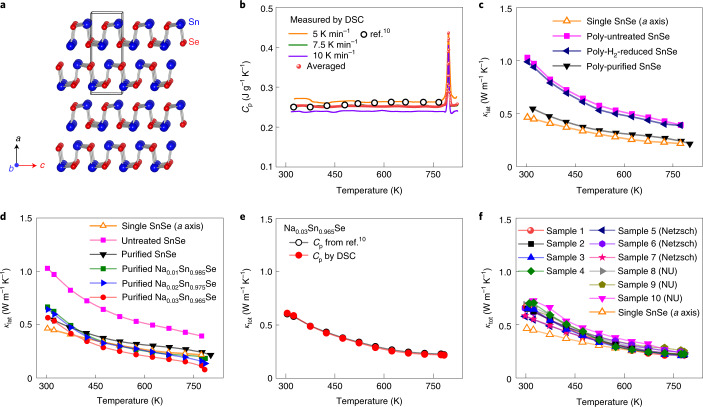
SnSe crystal structure and lattice, *κ*_lat_, and total thermal conductivities, *κ*_tot_, as a function of temperature for the undoped and Na-doped polycrystalline SnSe samples before and after the purification process. **a**, Room temperature crystal structure (*Pnma* space group) viewed down the *b* axis^9^: Sn atoms, blue; Se atoms, red. **b**, Temperature-dependent heat capacity (*C*_p_) measured by DSC for the purified Na_0.03_Sn_0.965_Se samples. Orange, green and purple solid lines denote the *C*_p_ recorded at the heating rate of 5, 7.5 and 10 K min^−1^, respectively. The averaged *C*_p_ values are represented by red circles, which are used to calculate the *κ*_tot_. *C*_p_ values derived from the previous work are included for comparison (black circles)^10^. **c**, *κ*_lat_ for the untreated, H_2_-reduced without Sn purification and purified SnSe samples. **d**, *κ*_lat_ for the Na_*x*_Sn_0.995–*x*_Se (*x* = 0.01, 0.02 and 0.03) samples in comparison with that for the untreated and purified SnSe samples. **e**, *κ*_tot_ of the Na_0.03_Sn_0.965_Se sample calculated using the *C*_p_ obtained by our DSC experiments (red circles) and derived from the previous works (black circles)^10^. **f**, The reproducibility of *κ*_tot_ for ten independently synthesized samples, cross-checked at SNU (samples 1–4), Netzsch Instruments (Netzsch, samples 5–7) and Northwestern University (NU, samples 8–10). *κ*_lat_ and *κ*_tot_ for a SnSe single crystal along the *a* axis are given for comparison^9^ in **c**,**d**,**f**. Polycrystalline samples were measured parallel to the SPS direction. Source data

The strong beneficial effect of our purification process is dramatically evident in the thermoelectric properties of polycrystalline SnSe. Because of the characteristic lamellar structure of SnSe (Fig. 4a), its thermoelectric properties are highly anisotropic^9^. Namely, polycrystalline and single-crystal samples exhibit the lowest thermal conductivity (*κ*) along the parallel direction of compaction (//) and along the crystallographic *a* axis^9^, respectively. Along these directions, we compare *κ* of our polycrystalline SnSe-based samples with the reported values for the undoped single-crystal sample^9^. To obtain accurate *κ*, we directly recorded the temperature-dependent heat capacity (*C*_p_) of the samples over the entire temperature range using differential scanning calorimetry (DSC). To ensure the credibility of data, we ran measurements for more than 20 samples. The *C*_p_ values taken at the three different heating rates of 5, 7.5 and 10 K min^−1^, respectively, unambiguously confirm that they are nearly constant outside the phase transition temperature of the Na_0.03_Sn_0.965_Se sample regardless of the heating rate (Fig. 4b). We averaged the obtained *C*_p_ values and then derived the *κ*. The averaged experimental *C*_p_ is comparable to the modelled value derived from the previous report^10^ over the entire range of temperature.

### Ultralow thermal conductivity

The purification process reduces lattice thermal conductivity (*κ*_lat_) for the polycrystalline SnSe sample and makes it comparable to that reported for single crystals over the entire temperature range (Fig. 4c). In contrast, when SnSe powder is treated only by the post H_2_-reduction without the Sn metal purification, the decrease in *κ*_lat_ is small. The *κ*_lat_ values for the untreated, H_2_-reduced and purified polycrystalline SnSe samples are roughly 1.03, 0.99 and 0.58 W m^–1^ K^–1^ at 300 K and roughly 0.39, 0.38 and 0.23 W m^–1^ K^–1^ at 773 K, respectively. This observation confirms that the application of a proper Sn purification procedure is essential to unveil the intrinsically ultralow *κ*_lat_ in SnSe-based thermoelectric materials.

Hole-doped Na_*x*_Sn_0.995−*x*_Se (*x* = 0.01–0.03) purified samples exhibit even lower *κ*_lat_ than the undoped polycrystalline and single-crystal SnSe samples. Their *κ*_lat_ decreases with the higher Na concentration because of slightly softened phonon frequency (Fig. 4d and Supplementary Fig. 6). The lowest *κ*_lat_ is roughly 0.17 (*x* = 0.01), 0.12 (*x* = 0.02) and 0.07 W m^–1^ K^–1^ (*x* = 0.03) at 783 K, in comparison with roughly 0.20 W m^–1^ K^–1^ at 973 K for the single-crystal SnSe along the *a* axis^9^. The observed value is one of the lowest *κ*_lat_ reported for bulk crystalline solids. In comparison, bulk polycrystalline CsAg_5_Te_3_ exhibits roughly 0.18 W m^–1^ K^–1^ at 727 K (ref. ^23^) and disordered thin films of lamellar WSe_2_, prepared by the vacuum deposition, give roughly 0.05 W m^–1^ K^–1^ at 300 K (ref. ^24^). The *x* = 0.03 sample shows the lowest total thermal conductivity (*κ*_tot_) among the series as the trend of *κ*_lat_ (Supplementary Fig. 7). Its *κ*_tot_ at 300 K is higher at roughly 0.65 than 0.46 W m^–1^ K^–1^ of the single-crystal SnSe sample. They show comparable *κ*_tot_ at the elevated temperatures, and the former exhibits a lower minimum of roughly 0.21 W m^–1^ K^–1^ at 783 K than roughly 0.23 W m^–1^ K^–1^ at 773 K of the latter.

The ultralow *κ* of Na_*x*_Sn_0.995−*x*_Se and SnSe samples up to 783 K is present before the sharp endothermic thermal event occurs, thus the phase transition has a negligible effect on the ultralow value of *κ*. Figure 4e demonstrates that the temperature-dependent *κ*_tot_ calculated by our DSC *C*_p_ and modelled *C*_p_ derived from the previous report^10^ are comparable from 300 to 783 K, confirming that *κ* is not underestimated by the modelled *C*_p_ in this temperature regime.

We prepared ten independent Na_0.03_Sn_0.965_Se specimens and cross-checked the reproducibility of the ultralow *κ* from two institutions, SNU and Northwestern University, and the manufacturer of Netzsch Instruments (Fig. 4f). The measurements on all specimens (four from SNU, three from Northwestern University and three from Netzsch) gave the uncertainty in *κ*_tot_ of less than roughly 10% in the temperature range 323–773 K.

### Charge transport properties

**Fig. 5:**
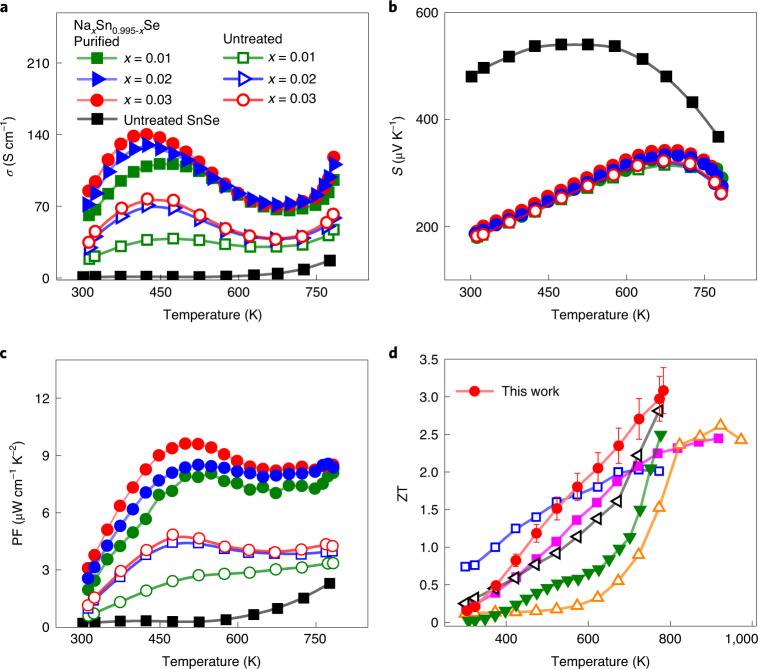
Thermoelectric properties of Na_*x*_Sn_0.995–*x*_Se before and after the purification process. **a**, Electrical conductivity (*σ*). **b**, Seebeck coefficient (*S*). **c**, Power factor (PF). **d**, ZT values of polycrystalline Na_*x*_Sn_0.995–*x*_Se developed in this work and current-state-of-the-art polycrystalline thermoelectrics, 2%Na-doped PbTe-8%SrTe^8^ (filled pink) and ball-milled and H_2_-reduced SnSe-5%PbSe doped with 1% Na^12^ (filled green) and single-crystal SnSe, undoped (p-type, open orange)^9^, single-crystal Na-doped (p-type, open blue)^10^ and single-crystal Br-doped SnSe^11^ (open black). Polycrystalline samples were measured parallel to the SPS direction. The typical uncertainty of 10% for ZT estimates is given. Source data

The effect of the purification process is seemingly marginal on electrical conductivity (*σ*) for the undoped polycrystalline SnSe samples (Supplementary Fig. 8a) because such samples have a very low carrier concentration (*n*_H_), for example, roughly 2.5 × 10^17^ and 2.0 × 10^17^ cm^−3^ at 300 K for the untreated and purified polycrystalline SnSe, respectively. In the doped Na_*x*_Sn_0.995−*x*_Se samples with *n*_H_ roughly 10^19^ cm^−3^, however, the purification process that minimizes surface SnO_*x*_ is key to achieving the enhanced Hall carrier mobility (μ_H_) (Supplementary Fig. 9) and *σ* over the full range of temperature (Fig. 5a). The *σ* markedly increases with the higher Na content in the temperature range 300–523 K. This leads to the enhanced thermoelectric performance of the samples in the low- to mid-temperature regime, a big improvement over previous polycrystalline SnSe thermoelectrics that suffer from low *σ* in that range, resulting in poor ZT values. The *x* = 0.03 sample shows the *σ* of 140 and 118 S cm^–1^ at 423 and 783 K measured parallel to the SPS direction, and 181 and 132 S cm^–1^ at the same temperatures perpendicular to the SPS direction.

The Seebeck coefficients (*S*) of the Na_*x*_Sn_0.995−*x*_Se samples are nearly the same along the parallel and perpendicular direction of SPS (Supplementary Fig. 10). Because of the higher hole concentration, the *S* values are lower than in the undoped SnSe samples (Fig. 5b). *S* slightly increases with the higher Na concentration consistent with the multi-band nature of the valence band in this material, which enhances the effective hole mass with higher hole concentrations as the Fermi level lowers to cross several valence bands according to our theoretical calculations (Supplementary Fig. 11) and the previous report^10^. Their *S* is slightly increased by our purification process making the Na doping more effective. For example, the maximum *S* for the *x* = 0.03 sample is +322 and +342 μV K^–1^ at 673 K before and after the purification process, respectively. The high reproducibility of *σ* and *S* values were confirmed using numerous independently synthesized specimens (Supplementary Fig. 12).

The simultaneously increased *σ* and *S* of the purified Na_*x*_Sn_0.995−*x*_Se samples result in the improved power factor (Fig. 5c) that trends higher with the rising Na concentration. The *x* = 0.03 sample exhibits power factors near 9 μW cm^–1^ K^–2^ in the wide range of temperature 473–783 K with a maximum of roughly 9.62 μW cm^–1^ K^–2^ at 498 K parallel to the SPS direction. The maximum power factor is roughly 12.06 μW cm^–1^ K^–2^ at 473 K perpendicular to the SPS direction, which is the highest value reported for polycrystalline SnSe-based materials.

### Thermoelectric figure of merit

The purification process concurrently enhances *σ* and *S*, and decreases *κ*_tot_ for the Na_*x*_Sn_0.995−*x*_Se samples, leading to an extraordinarily high thermoelectric figure of merit ZT. It increases with higher Na concentration. The *x* = 0.03 sample exhibits the maximum ZT (ZT_max_) roughly 3.1 at 783 K, which is the highest reported for any thermoelectric system. This ultrahigh thermoelectric performance is attained below the phase transition temperature as observed in our DSC results (Fig. 4b), affirming no overestimation of ZT by the phase transition. In comparison, p- and n-type SnSe single crystals exhibit a ZT_max_ of roughly 2.6 at 923 K (ref. ^9^) and 2.8 at 773 K (ref. ^11^), respectively (Fig. 5d). Among the highest performance polycrystalline thermoelectric systems have been PbTe-8%SrTe doped with 2% Na (ZT_max_ roughly 2.5 at 923 K, ref. ^8^) and ball-milled and H_2_-reduced SnSe-5%PbSe doped with 1% Na (ZT_max_ roughly 2.5 at 773 K, ref. ^12^). ZT for Na_0.03_Sn_0.965_Se already exceeds unity above 473 K, at which temperature regime few materials show comparable performance. It exhibits a record-high average ZT^25^ roughly 2.0 from 400 to 783 K (Supplementary Fig. 13). The deviation in temperature-dependent ZT values on consecutive heating and cooling cycles is less than 10%, indicating the prospect of stable operation from 300 to 783 K for thermoelectric power generation (Supplementary Figs. 14 and 15).

We conclude that a trace of SnO_*x*_ in the starting tin metal reagent, used to prepare SnSe samples, has persistently concealed the intrinsic charge and thermal transport properties of SnSe and prevented the full thermoelectric performance from being realized. When properly purified and doped using the methods described above, polycrystalline SnSe exhibits an extraordinarily high ZT of roughly 3.1, outperforming any other bulk thermoelectric systems. The ultrahigh thermoelectric performance indeed originates from the intrinsic crystal chemistry of this simple yet remarkable binary compound SnSe, and this bodes well for the future development of this material to affect power generation applications from waste heat. This revelation has broader implications of how other systems need to be handled in the future and calls for the re-examination of synthesis and sample preparation processes for extensively studied thermoelectric systems, especially those containing tin.

## Methods

### Note

All procedures for the synthesis and sample preparations were strictly carried out in an Ar-filled glovebox (99.99% purity), in which the levels of moisture and oxygen are kept at 0 and less than 1 ppm, respectively, unless noted otherwise. When samples were transported from the glovebox for measurements and compaction processes, they were properly protected under a mobile Ar flowing system.

### Reagents

The following starting reagents were used as received unless noted otherwise: Se shot (99.999%, 5N Plus) and Na piece (99.9%, Sigma-Aldrich). As-received Sn chunk (99.999%, American Elements) was used to synthesize ‘untreated’ SnSe-based materials as control samples. It was purified by our melting-purification process as described below to eliminate surface tin oxides, and was used to synthesize ‘purified’ SnSe-based materials.

### Purification of Sn

As-received Sn chunks were apparently silvery. They were placed on a graphite sheet prewashed with ethanol, and were heated at 473 K, which is near the melting point of Sn at roughly 505 K, for 6 h under a 4% H_2_/96% Ar with a flow rate of 200 ml min^–1^. A change in their surface colour and lustre was invisible. The resulting Sn chunks were loaded into a carbon-coated and evacuated fused-silica tube (roughly 10^–4^ Torr). The tube was heated at 1,273 K for 6 h, followed by cooling to room temperature. Ash-like black residues formed at the top and surface of the Sn ingot. They were identified as tin oxides by Fourier transformed far-infrared absorption spectroscopy. They were scraped out of the Sn ingot. The same melting-purification process was repeated three times at 873–723 K until the black residues were no longer observed.

### Synthesis

Purified and untreated materials with the nominal compositions Na_*x*_Sn_0.995–*x*_Se (*x* = 0.01–0.03) and SnSe as a reference were synthesized by reacting stoichiometric mixtures of proper starting reagents. They were loaded in carbon-coated and evacuated fused-silica tubes (roughly 10^–4^ Torr), and were heated at 1,223 K for 12 h, followed by quenching to ice water. The obtained ingots were further annealed at 773 K for 48 h and were cooled naturally to room temperature. The weight of typical ingots was approximately 13 g. They were pulverized by hand-grinding, and were subsequently purified at 613 K for 6 h under a 4% H_2_/96% Ar with a flow rate of 200 ml min^−1^.

### Compacting powders

The resulting powders were loaded in a BN-coated graphite die and were cold-pressed manually in an Ar-filled glovebox. To avoid any possible oxidation of a sample, the loaded die was tightly sealed in a plastic zipper bag and taken out of the glovebox. It was transported from the chamber of the glovebox to the adjacently placed SPS system (SPS-211Lx, Fuji Electronic Industrial Co.) under a mobile Ar (99.99%) flowing system. Powder samples in the die were densified at roughly 783 K for 5 min under an axial pressure of 50 MPa in a vacuum of roughly 1.4 × 10^−2^ Torr using SPS. All SPS-processed samples show relative densities of roughly 96%.

### Powder X-ray diffraction

We carried out X-ray diffraction analysis on a SmartLab Rigaku X-ray diffractometer with Cu Kα (*λ* = 1.5418 Å) graphite-monochromatized radiation operating at 40 kV and 30 mA at room temperature. The patterns measured parallel and perpendicular to the pressing direction of the SPS-processed ingots for purified and untreated SnSe and Na_0.03_Sn_0.965_Se samples are given in Supplementary Figs. 16 and 17.

### TOF–SIMS

TOF–SIMS experiments were carried out on a Physical Electronics TRIFT III spectrometer. The SPS-processed samples were polished with Buehler Ecomet III Tabletop Polisher/Grinder to prepare a smooth surface. Subsequently, they were sputtered with a 5 keV Ar ion beam for 5 min in SIMS chamber to expose the GB. During this process, omnipresent H_2_O even in an ultrahigh vacuum chamber was ionized to give H^+^, which then attached to surface tin oxides to form SnOH^+^. Accordingly, to examine the distribution of surface tin oxides, the SnOH^+^ ion mapping images were collected for 10 min. The primary ion source of SIMS is gallium beam with 25 keV energy. The measurements were conducted in NUANCE-Keck-II centre of Northwestern University.

### Hall measurements

The Hall coefficients (*R*_H_) were obtained by the Van der Pauw method on a Lake Shore HMS8407 Hall effect measurement system in a magnetic field of 1.5 T and 3 mA excitation current. The hole carrier concentration (*n*_H_) and hole mobility (*μ*_H_) were accessed by the formulas, *n*_H_ = 1/(e*R*_H_) and *μ*_H_ = *R*_H_*σ*, respectively.

### Electrical and thermal transport property measurements

The obtained SPS-processed pellets were cut and polished into a rectangular shape with a length of 13 mm and thickness of roughly 2 mm under a N_2_ atmosphere (99.99% purity) (Supplementary Fig. 18). The electrical conductivity and Seebeck coefficient were measured simultaneously under an Ar atmosphere from room temperature to 823 K using a Netzsch SBA 458 Nemesis system. A Netzsch LFA 457 MicroFlash instrument was used to record the thermal diffusivity of the samples coated with graphite. The typical samples are disc shaped with a diameter of 8 mm and thickness ranging from 1 to 2 mm. To confirm the reproducibility of ultralow thermal conductivity of the Na_0.03_Sn_0.965_Se samples, the thermal diffusivity was cross-checked at Northwestern University and Netzsch Instruments (Korea) using LFA 457 and 467 instruments, respectively (Supplementary Figs. 19–21). The thermal conductivity was calculated from the formula *κ*_tot_ = *DC*_p_*ρ*, where *D* is the thermal diffusivity, *C*_p_ is the heat capacity, which was directly measured using the DSC technique, and *ρ* is the mass density of the specimens. The *ρ* value used was obtained by their geometrical dimensions and masses, which is nearly the same as that by the Archimedes method. The density values used are given in Supplementary Table 1. The total thermal conductivity *κ*_tot_ is the sum of the lattice (*κ*_lat_) and electronic thermal (*κ*_ele_) conductivities. *κ*_ele_ is proportional to the electrical conductivity (*σ*) according to the Wiedemann–Franz relation (*κ*_ele_ = *L*σ*T*), where *L* is the temperature-dependent Lorenz number and *T* is the absolute temperature. The *κ*_lat_ value was calculated by subtracting the *κ*_ele_ from the *κ*_tot_ value by the relation *κ*_lat_ = *κ*_tot_ – *κ*_ele_. Average ZT (ZT_ave_) was calculated using the following equation^25^:

$${\mathrm{ZT}}_{{\mathrm{ave}}} = \frac{{\mathop {\smallint }\nolimits_{T_{{\mathrm{cold}}}}^{T_{{\mathrm{hot}}}} {\mathrm{ZTd}}T}}{{T_{{\mathrm{hot}}} - T_{{\mathrm{cold}}}}}$$

where *T*_hot_ and *T*_cold_ represent the temperature at the hot and cold sides, respectively.

### Heat capacity measurements

The temperature-dependent heat capacity (*C*_p_) was experimentally recorded by differential scanning calorimeter (DSC Polyma 214, Netzsch). To minimize the error, samples were cut into a cube with dimensions of roughly 2 × 2 × 2 mm^3^. Because Na easily reacts with typical Al_2_O_3_ or Al crucibles, Pt crucibles were used. Before the measurement, the blank crucible was heated to 823 K at least twice under a high-purity argon (99.999%) flow to remove any possible residual water and physisorbed O_2_. Afterwards, a standard sapphire disc with a diameter of 4 mm and a thickness of 0.25 mm was loaded into the crucible and measured up to 823 K. Subsequently, the standard sapphire was taken out, and the Na_0.03_Sn_0.965_Se sample was placed in the same crucible. The loaded crucible was purged with a high-purity argon flow for 30 min to ensure a dry and air-free atmosphere before the measurement. The *C*_p_ was extracted by comparing the signal difference between the reference sapphire and sample^26^.

### STEM

STEM specimens were excised from the GB using a dual-beam scanning electron microscope/focused ion beam (Helio NanoLab 650, FEI) system using gallium ion milling. Before the ion milling, the surface of specimens was protected with carbon coating by sputtering. Structures and chemical compositions around GBs were analysed using a spherical aberration-corrected JEM ARM-200F microscope (Cold FEG Type, JEOL) equipped with an SDD type energy-dispersive X-ray spectroscopy (EDS) detector (Solid Angle 0.9-sr, X-MaxN 100TLE, Oxford) at 200 kV installed at the National Centre for Inter-University Research Facilities in SNU. In HAADF–STEM images, the point-to-point resolution was approximately 80 pm after correcting the spherical aberration, and the angular range of the annular detector used was from 68 to 280 mrad. All STEM images were recorded using a high-resolution CCD detector with a 2,000 × 2,000-pixel device in the GIF-QuantumER imaging filter (Gatan). For STEM–EDS investigation, chemical maps were acquired with a probe size of 0.13 nm and a probe current of 40 pA.

### APT

APT needle-shaped specimens were prepared using a dual-beam scanning electron microscope/focused ion beam (Helios NanoLab 650, FEI) following the site-specific ‘lift-out’ method^27^. The specimens were measured in a local electrode atom probe (LEAP 4000 X Si, Cameca) with voltage- and laser-assisted evaporation modes for the Sn reagent and SnSe samples, respectively. For a voltage mode, a voltage pulse with a repetition rate of 200 kHz and pulse fraction of 20% was used. The detection rate was five ions per 1,000 pulses (0.5%) on average. The base temperature of specimen was 30 K. For a laser mode, a 5 pJ ultraviolet (wavelength, 355 nm) laser with 10 ps pulse and a 200 kHz repetition rate was used. The detection rate was one ion per 100 pulses (1%) on average. For both modes, the base temperature was 40 K and the ion flight path was 160 mm. The detection efficiency was limited to 50% due to the open area of the microchannel plate. The APT data were processed using the software package IVAS v.3.8.0 (ref. ^18^).

### Calculations for phonon band structure and Grüneisen parameters

Phonon band structure and Grüneisen parameters were calculated within quasi-harmonic approximations based on DFT calculations. They have been calculated for pristine SnSe previously^9^. In this work, we further calculated them for the optimally hole-doped system, namely, Na_0.03_Sn_0.97_Se. To obtain accurate force constant matrix, we used a 2 × 2 × 2 supercell for Na_0.03_Sn_0.97_Se with 64 atoms, and accordingly the supercell accommodates 512 atoms in total. For better comparison, we also considered the same size of supercell for pristine SnSe. DFT force calculations were performed with a plane wave set of 350 eV energy cutoff, gamma point *k*-space sampling and PBEsol exchange functional^28^, and they were forced to converge until the largest component of atomic force becomes smaller than 10^–8^ eV Å^–1^. To evaluate the Grüneisen parameters defined by the relation $\gamma _i = -\frac{V}{{\omega _i}}\frac{{\partial \omega _i}}{{\partial {\mathrm{V}}}}$, where *V* is the volume of unit cell and *ω*_*i*_ is frequency of *i*-th phonon mode, we considered three sets of phonon dispersion relation with different volumes, namely, 0.99, 1.00 and 1.01 times the optimized volume of the unit cell. Calculation results are presented in Supplementary Fig. 6.

### Calculations for electronic band structure

To understand the enhanced Seebeck coefficient by Na doping, we obtained electronic band structures for hole-doped Na_0.03_Sn_0.97_Se and pristine SnSe using DFT calculations with plane wave basis set with 350 eV energy cutoff, 4 × 4 × 4 *k*-space sampling, and SCAN + rVV10 functional^29^. We used a $2\sqrt 2 \times 2\sqrt 2$× 1 supercell, and the lattice parameters and internal coordinates were fully optimized. On Na doping, the lattice dimension decreases by about 0.5% along the *b* and *c* axes and increases by about 0.3% along the *a* axis.

It should be noted that the recent investigation by angle-resolved photon emission spectroscopy (ARPES) for SnSe clearly shows the emergence of pudding mould-type bands near valence band maximum^30^, which is highly important for achieving high power factor within the band convergence strategy. However, many previous DFT studies for SnSe using semilocal exchange-correlation functional such as Perdew–Burke–Ernzerhof (PBE) generalized gradient approximation could not reproduce band structures observed by the ARPES appropriately. In this work, we found that such band dispersions in SnSe seen by the ARPES experiments can be well reproduced by SCAN + rVV10 functional. Accordingly, we applied the same method to hole-doped Na_0.03_Sn_0.97_Se. We considered a 64-atom-containing supercell for both SnSe and Na_0.03_Sn_0.97_Se. Band structures are evaluated along a high-symmetric line in *k*-space. For density of states, we used a denser 10 × 10 × 10 regular mesh. Calculation results are given in Supplementary Fig. 11.

## Online content

Any methods, additional references, Nature Research reporting summaries, source data, extended data, supplementary information, acknowledgements, peer review information; details of author contributions and competing interests; and statements of data and code availability are available at 10.1038/s41563-021-01064-6.

## Supplementary information

Supplementary Information Supplementary Note, Figs. 1–21, Table 1 and refs. 1–12.

## Data Availability

The datasets for Figs. 1–5 are available in the source data section. Additional information is available from the authors on request. Source data are provided with this paper.

## Source data

Source data Fig. 1 Thermal conductivity and ZT comparison.
Source data Fig. 2 Figure 2c,d; TOF–SIMS results.
Source data Fig. 3 Figure 3g, h, l, m; APT results.
Source data Fig. 4 Figure 4b–f; Thermal transport properties.
Source data Fig. 5 Figure 5a–d; Thermoelectric properties.

## Code availability

The computer codes that support the findings of this study are available from the corresponding author on reasonable request.
