# Supplementary material for: Polycrystalline SnSe with a thermoelectric figure of merit greater than the single crystal
Source: Nat Mater. 2021 Aug 2;20(10):1378–84. doi: 10.1038/s41563-021-01064-6 (PMC8463294; doi:10.1038/s41563-021-01064-6)
Supplement: Supplementary file 1 — Supplementary Note, Figs. 1–21, Table 1 and refs. 1–12. [file 41563_2021_1064_MOESM1_ESM.pdf]

nature portfolio

https://doi.org/10.1038/s41563-021-01064-6

Supplementary information

Polycrystalline SnSe with a thermoelectric figure of merit greater than the single crystal

In the format provided by the authors and unedited

Supplementary information for

Polycrystalline SnSe with a thermoelectric figure of merit greater than the single-crystal

Chongjian Zhou[1,10], Yong Kyu Lee[1,10], Yuan Yu[2,10], Sejin Byun[1,3], Zhong-Zhen Luo[4], Hyungseok Lee[1,3], Bangzhi Ge[1], Yea-Lee Lee[5], Xinqi Chen[6], Ji Yeong Lee[7], Oana Cojocaru-Mirédin[2], Hyunju Chang[5], Jino Im[5], Sung-Pyo Cho[8], Matthias Wuttig[2], Vinayak P. Dravid[9], Mercouri G. Kanatzidis[4,9,*], In Chung[1,3*]

[1]School of Chemical and Biological Engineering, and Institute of Chemical Processes, Seoul National University, Seoul 08826, Republic of Korea.

[2]Institute of Physics (IA), RWTH Aachen University, 52056 Aachen, Germany

[3]Center for Correlated Electron Systems, Institute for Basic Science (IBS), Seoul 08826, Republic of Korea.

[4]Department of Chemistry, Northwestern University, Evanston, IL 60208, USA.

[5]Chemical Data-Driven Research Center, Korea Research Institute of Chemical Technology, Daejeon 34114, Republic of Korea.

[6]Department of Mechanical Engineering, Northwestern University, Evanston, IL 60208, USA.

[7]Advanced Analysis Center, Korea Institute of Science and Technology, Seoul 02792, Republic of Korea

[8]National Center for Inter-University Research Facilities, Seoul National University, Seoul 08826, Republic of Korea

[9]Department of Materials Science and Engineering, Northwestern University, Evanston, IL 60208, USA.

[10]These authors contributed equally to this work

*Correspondence to: m-kanatzidis@northwestern.edu (M.G.K.); inchung@snu.ac.kr (I.C.)

Table of Contents

This Supplementary Information file archives:

1. Supplementary note describes the calculation details employed through this article.

2. Supplementary Figs. 1-21 are chronologically cited in the main text.

Supplementary Figs. 1-3 present the purification process of Sn and the corresponding composition analysis of the residuals.

Supplementary Figs. 4-21 show the additional experiment data and theoretical calculation results for $Na_xSn_{0.995-x}Se$.

3. Supplementary Table 1 shows the density of purified samples.

4. References.

Supplementary Note

Calculations for formation energy of oxygen impurity and phase stability. The formation energies of oxygen impurities were calculated using an octuple super cell of SnSe. The lattice parameters of pristine SnSe were fully relaxed to be a = 11.49 Å, b = 4.12 Å, and c = 4.53 Å, while the internal coordinate of three possible configurations of oxygen impurities such as including O replacing Sn (O_{Sn}), O replacing Se (O_{Se}), and O at interstitial sites in the SnSe crystal lattice (O_{Int}) were optimized within the fixed lattice constant of pristine SnSe by the density functional theory (DFT) calculations. Plane wave basis set with 350 eV cut off energy and the projector augmented wave method[1] were utilized to describe wave functions and potentials. For the accurate description of exchange correlation functional and van der Waals interaction, we employed SCAN+rVV10 functional[2] which is implemented in Vienna Ab initio Simulation Package[3,4]. Formation energies of each configuration were calculated with the following formula:

$$\Delta H_{D,q}(E_F,\mu)=\left[E_{D,q}-E_H\right]+q(E_V+\Delta E_F)+\sum_\alpha n_\alpha\left(\mu_\alpha^0+\Delta\mu_\alpha\right)+\varepsilon_{corr}$$

The first term of right-hand side of the equation is the total energy difference between defected system with charge q and pristine host material. The second and third terms stand for the free energy gain (or loss) from Fermi energy and chemical potential, respectively, where E_V is the highest energy level of occupied states of electron, ΔE_F is the Fermi level referenced to E_V, n_α is the number of atom when α is removed (+1, +2, ..) or added (–1, –2, …) to host system upon the defect formation, μ_α^0 is the total energy of reference phase of α, and $\Delta\mu_\alpha$ is the relative chemical potential α that varies with synthesis condition. The last term is a correction term including finite cell corrections[5]. Supplementary Fig. 4a presents the formation energy of three different configurations of oxygen impurities in SnSe as a function of Fermi level under the oxygen-rich

condition. The set of corresponding chemical potentials is determined by free energies of competing phases consisting of Sn, Se, and O, which results in the phase stability map shown in Supplementary Fig. 4c. From the calculated formation energies of oxygen impurities, the concentration of oxygen which can be soluble into the lattice is evaluated as shown in Supplementary Fig. 4b.

The facile formation of surface SnO_x can be understood by density functional theory calculations. If oxygen is introduced to the interior of the SnSe lattice (rather than the surface), the possible defects are O replacing Sn (O_{Sn}), O replacing Se (O_{Se}), and O at interstitial sites in the SnSe lattice (O_{Int}). Their defect formation energy is calculated to be larger than 1.0 eV (Supplementary Fig. 4a) under the oxygen-rich condition (indicated by a red dot in Supplementary Fig. 4c). This result indicates that it is unlikely to form oxygen defects in the SnSe crystal structure as the maximum atomic concentration of oxygen impurity present in the SnSe lattice cannot exceed 10^{14} cm^{-3} in the temperature range of 300–800 K (Supplementary Fig. 4b). Hence, even a trace amount of oxygen present in the starting reagents and during the sample preparation processes can form sub-stoichiometric SnO_x on the surface of SnSe powders. The presence of this nominal SnO_x layer acts as a barrier to preclude oxygen diffusion/reaction to reach the interior of the SnSe grains. Supplementary Fig. 4c presents the calculated phase stability map of SnSe and oxygen-containing competitors as a function of chemical potential for oxygen ($\Delta\mu_O$). At small enough $\Delta\mu_O$ of less than –3.33 eV, only SnSe exists. As $\Delta\mu_O$ exceeds –3.33 eV, SnO_x precipitates first. Thus, DFT calculations support the observation of a high degree of SnO_x at grain boundaries in polycrystalline SnSe samples.

Supplementary Figs. 1-21

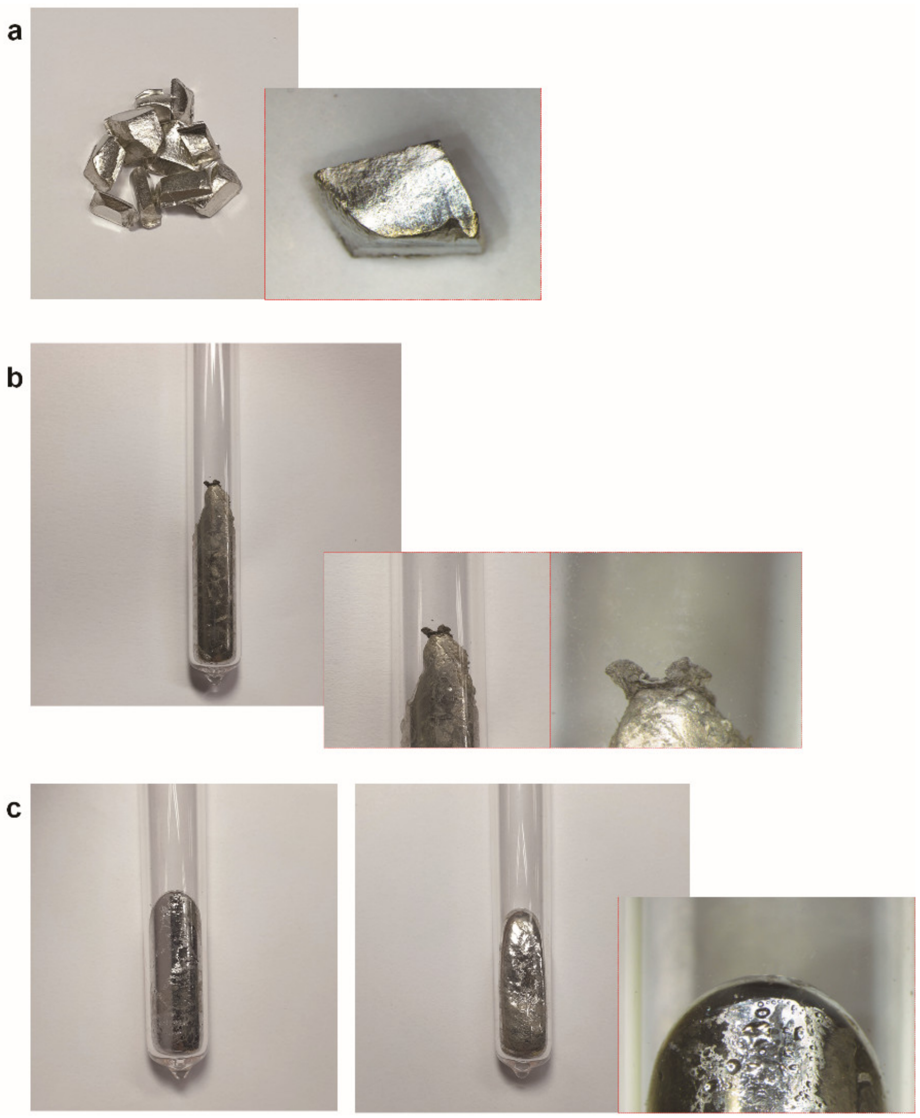

Supplementary Fig. 1. a, As-received Sn chunks. **b,** Typical images of Sn ingot after H_2-chemical reduction and subsequent melting-purification at 1273 K for 6 h. It is clearly observed that ash-like black residues formed on the top and whole surface of Sn ingot. **c,** After the repetition of the melting-purification process three times, the black residues were invisible any more.

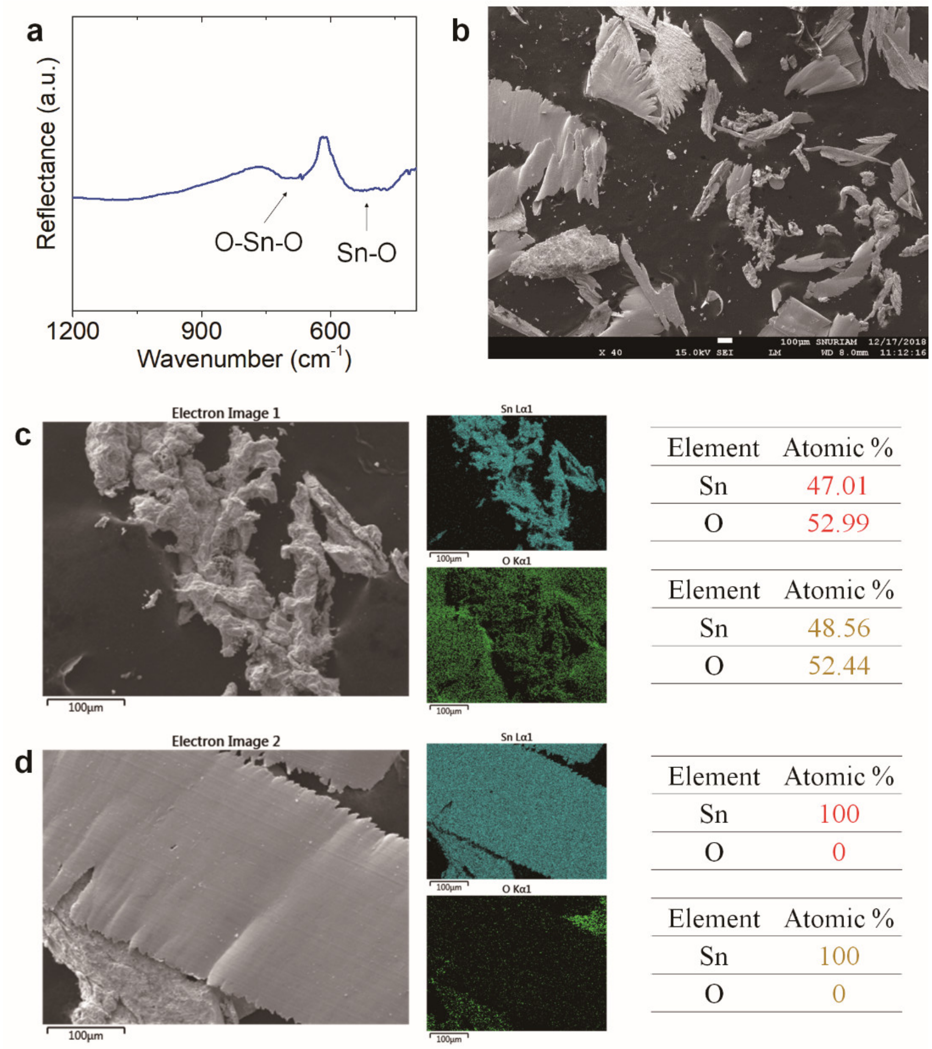

Supplementary Fig. 2. Spectroscopic and elemental analysis on the ask-like black residues. The black residues, shown in Supplementary Fig. 1b, were carefully scraped from the top and surface of the Sn ingot after the first melting-purification process. **a,** Fourier-transformed far-infrared spectrum of the ash-like black residues. The peaks in the spectrum arise from the presence of Sn-O bonds. The bands around 480 cm^{-1} and 540 cm^{-1} are attributed to asymmetric and symmetric stretching vibration of O-Sn-O, respectively. The band at 680 cm^{-1} can be assigned to the asymmetric Sn-O-Sn stretching mode[6–9]. **b,** Scanning electron microscopy (SEM) image of the ash-like black residues with the slices of Sn, which were scraped from Sn ingot. **c,** SEM image, EDS elemental mapping, and composition analysis of the ash-like black residues, confirming they are SnO_x. **d,** SEM image, EDS elemental mapping, and composition analysis of Sn slices, confirming the negligible concentration of SnO_x in the purified Sn ingot.

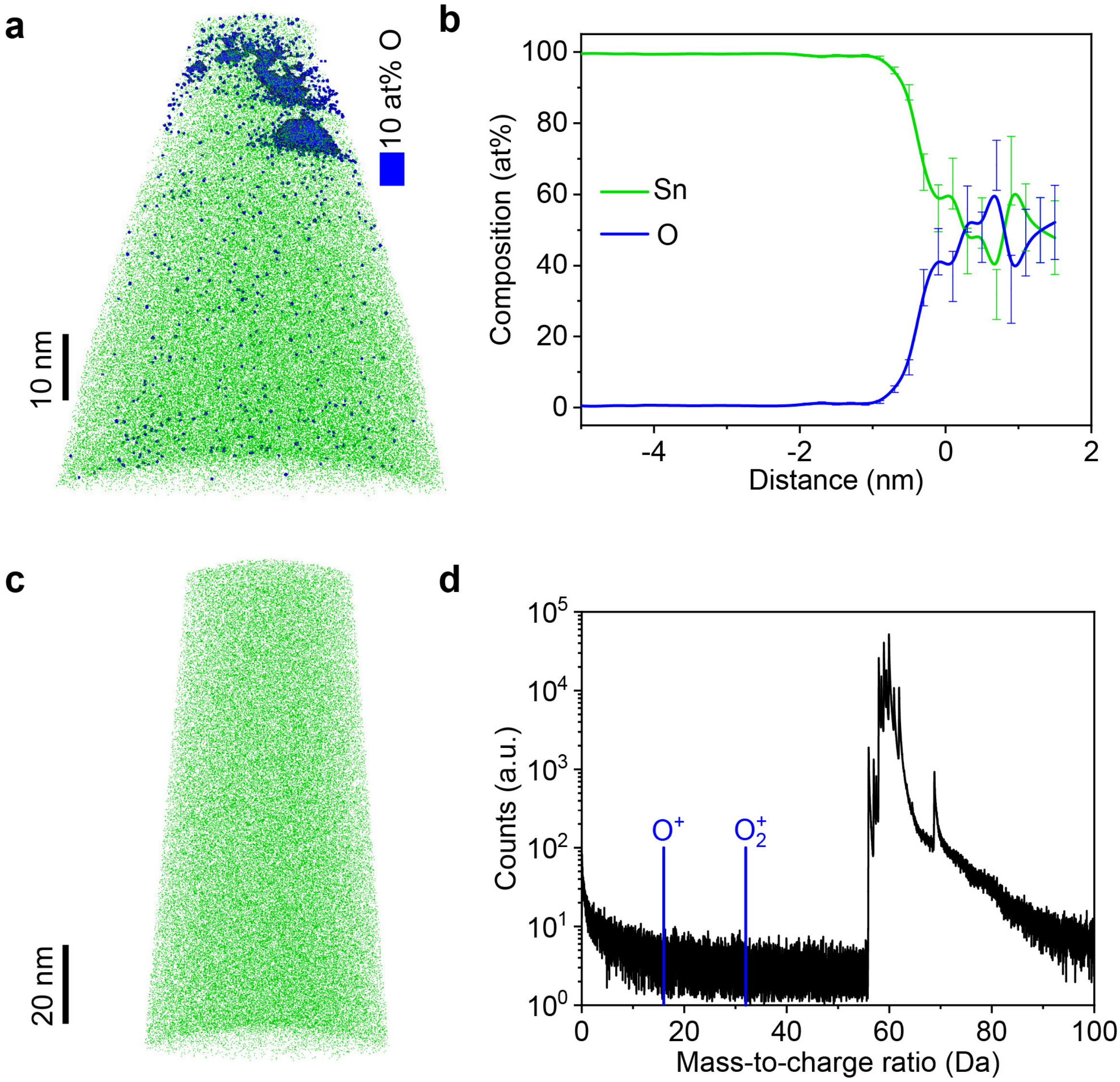

Supplementary Fig. 3. Composition analysis of element Sn by atom probe tomography. a. Three-dimensional APT reconstruction of the specimen lifted out from the surface of Sn ingot after the first melting-purification process. The Sn and O atoms are mapped as green and blue dots, respectively. A high degree of O cluster is clearly observed (denoted by navy isosurface of 10 at % O). **b**. Proximity histogram displaying the concentration profile of Sn and O atoms across an individual O-rich region. The histogram confirms O heavily aggregates within commercially available Sn, resulting in the formation of SnO_x. **c**. Three-dimensional APT reconstruction of the purified Sn. The O atoms were not detected, confirming our purification process successfully removed SnO_x in raw materials. **d.** The mass-to-charge ratio spectrum for the purified Sn confirming the absence of signals from O atoms as indicated by the blue solid lines.

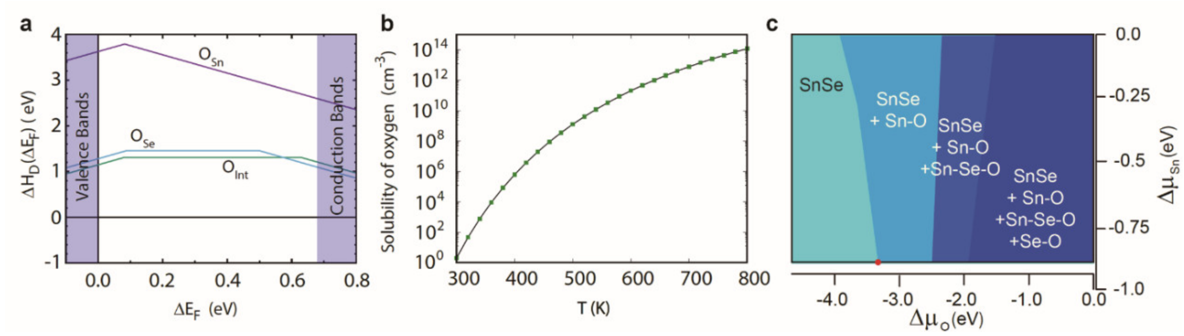

Supplementary Fig. 4. The density functional theory calculations for the formation of surface tin oxides. a, The diagram of calculated defect formation energy (ΔH_D) as a function of Fermi energy (ΔE_F) for three possible defects: oxygen replacing tin (O_{Sn}), oxygen replacing selenium (O_{Se}), and oxygen at interstitial sites in the SnSe lattice (O_{Int}). The energy level of the valence band maximum (VBM) is set to zero. The regions below VBM and above conduction band minimum are depicted in violet color. **b,** The calculated solubility of oxygen in the SnSe lattice in the temperature range of 300-800 K, showing that the maximum concentration of oxygen impurity present in the SnSe lattice cannot exceed 10^{14} cm^3. **c,** Phase stability map of SnSe and competitors with oxygen as a function of chemical potential for oxygen ($\Delta\mu_O$).

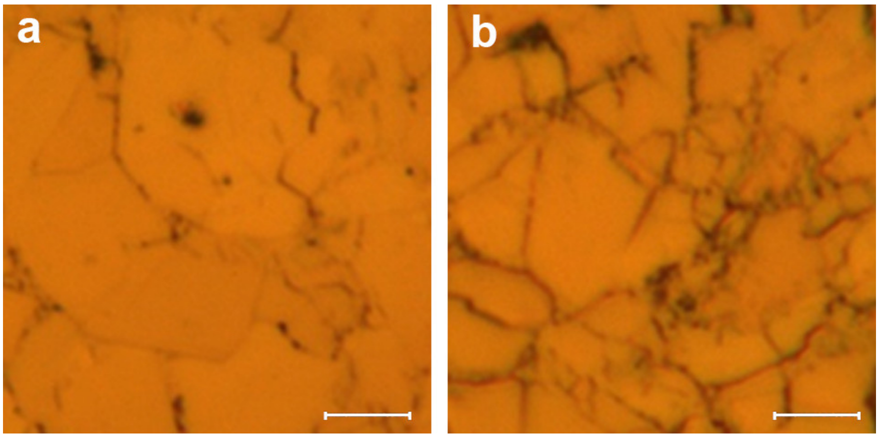

Supplementary Fig. 5. The corresponding optical images taken at the same area for the TOF-SIMS images in the main text. a, 'Untreated' polycrystalline SnSe. **b,** 'Purified' polycrystalline SnSe. The scale bar is 10 μm.

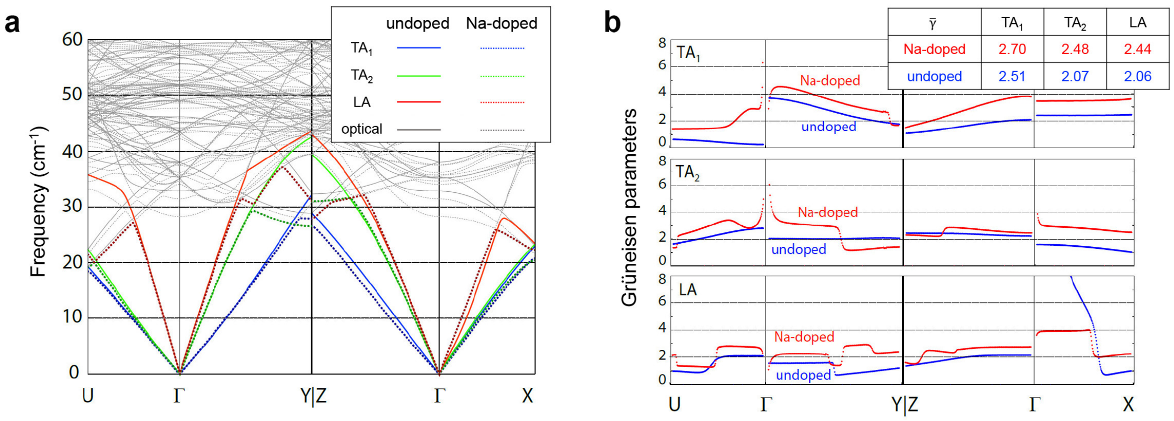

Supplementary Fig. 6. Theoretical calculations for phonon dispersions and Grüneisen parameters. a, Calculated phonon dispersion for pristine and Na doped SnSe. TA, Transverse acoustic phonon scattering branches; LA, longitudinal acoustic phonon scattering branch. **b,** Grüneisen parameters: inset, the average Grüneisen parameters along the TA_1, TA_2, and LA. We investigated phonon dispersions and Grüneisen parameters for undoped and 3% Na-doped SnSe using first-principles DFT phonon calculations within the quasi-harmonic approximation. Na-doping slightly softens phonon frequency in **a**. Especially, the lowest energy transverse acoustic mode (TA_1) is prominently softened along the Γ-Z Brillouin zone direction with the decrease in phonon velocity of TA_1 mode from 1548 to 1305 $m \cdot s^{-1}$. Grüneisen parameters measure the strength of the bonding anharmonicity. The average Grüneisen parameters (γ) for all the acoustic phonon modes increase by Na doping from 2.51, 2.07, and 2.06 to 2.70, 2.48, and 2.44 for TA_1, TA_2, and LA modes, respectively in **b**. Given κ_{lat} proportional to γ^{-2}, the benefit from enriched anharmonicity by Na doping is greater than the accompanying increase in κ_{ele}, thereby lowering κ_{tot} for Na-doped SnSe.

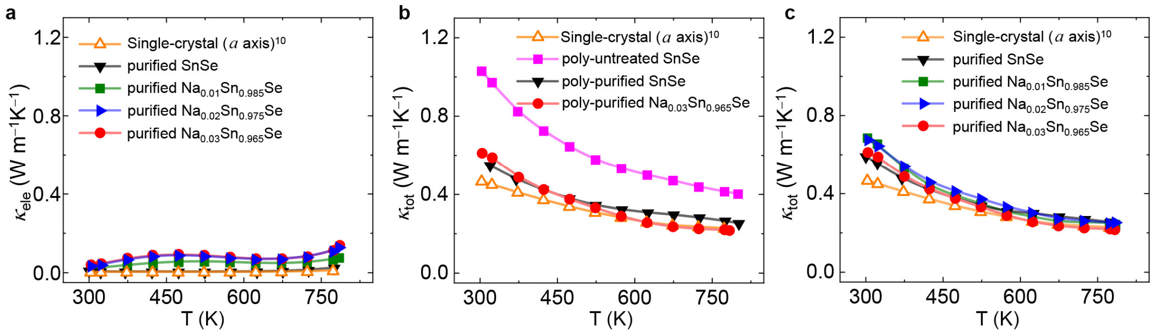

Supplementary Fig. 7. **Thermal conductivities of the purified $Na_xSn_{0.995-x}Se$ and SnSe as a function of temperature. a,** Electronic thermal conductivity, κ_{ele}. **b,** Comparison of total thermal conductivity, κ_{tot}, for the purified $Na_{0.03}Sn_{0.965}Se$ and untreated and purified SnSe. **c,** Comparison of κ_{tot} for purified $Na_xSn_{0.995-x}Se$ and SnSe. κ_{lat} and κ_{tot} for SnSe single-crystal along the *a* axis are given from the previous report for comparison[10]. Data for polycrystalline samples were measured along the SPS direction. The untreated sample was synthesized using as-received starting Sn reagent. The purified samples were synthesized using starting Sn reagent treated by our purification process.

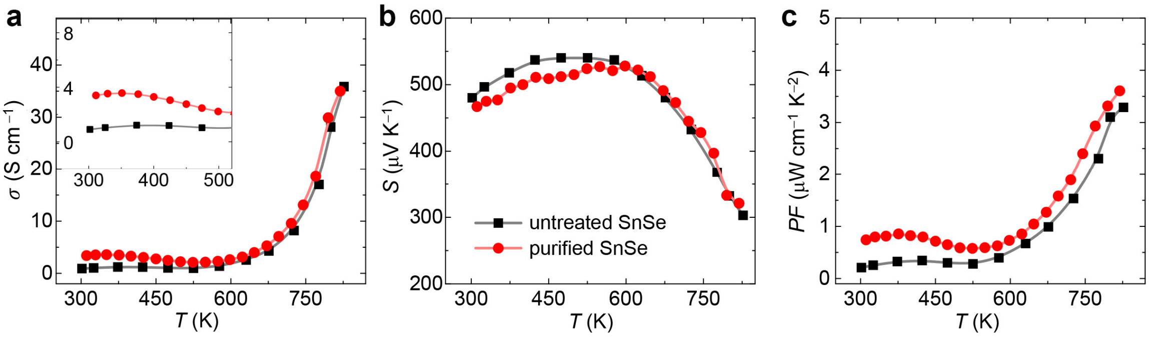

Supplementary Fig. 8. Charge transport properties as a function of temperature for polycrystalline SnSe before and after the purification process. a, Electrical conductivity, σ. Inset, σ in the low temperature range is magnified. **b,** Seebeck coefficient, S. **c,** Power factor, PF. Data were measured along the SPS direction. The untreated sample was synthesized using as-received starting Sn reagent. The purified sample was synthesized using starting Sn reagent treated by our purification process.

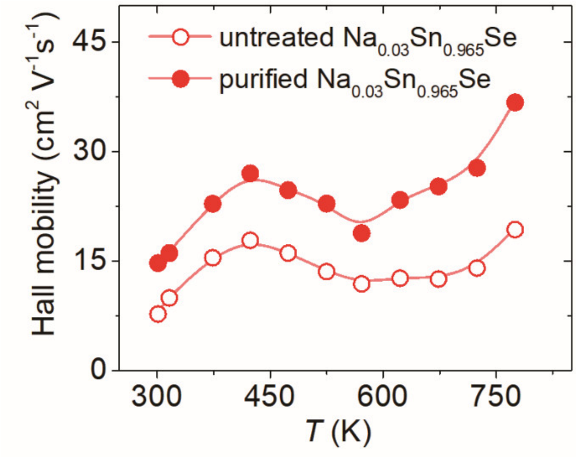

Supplementary Fig. 9. Hall mobility for the untreated and purified $Na_{0.03}Sn_{0.965}Se$ samples as a function of temperature.

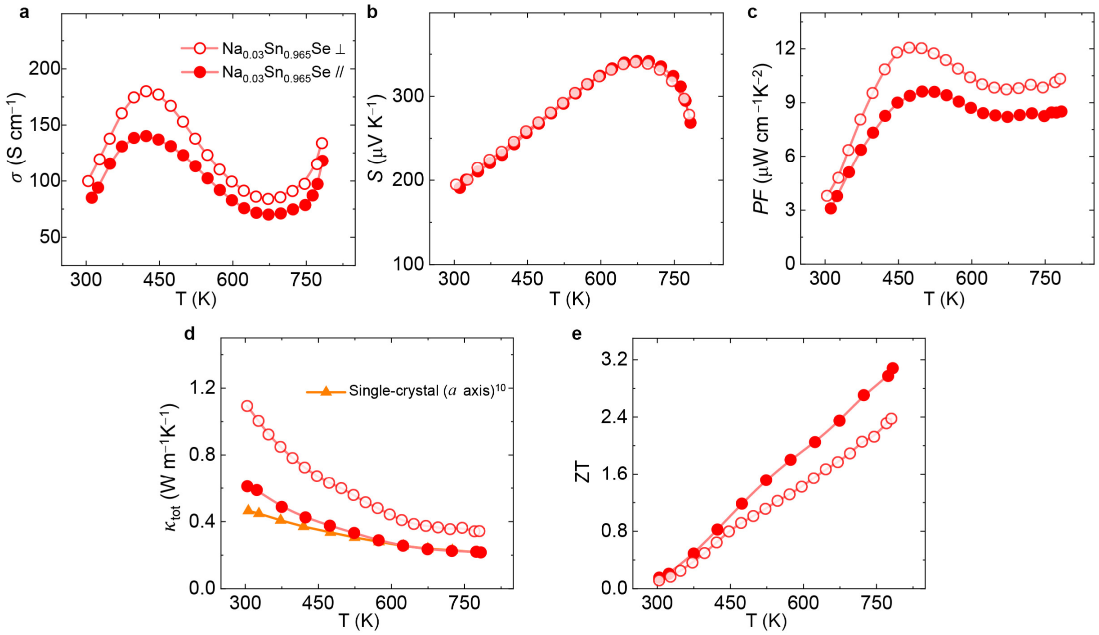

Supplementary Fig. 10. Comparison of thermoelectric properties as a function of temperature for the purified $Na_{0.03}Sn_{0.965}Se$ sample parallel, //, and perpendicular, ⊥, to the SPS direction. a, Electrical conductivity, *σ*. **b,** Seebeck coefficient, *S*. **c,** Power factor, *PF*. **d,** Total thermal conductivity, κ_{tot}. **e,** ZT values. κ_{tot} for single-crystal SnSe along the *a* axis from the previous report[10] is given for comparison.

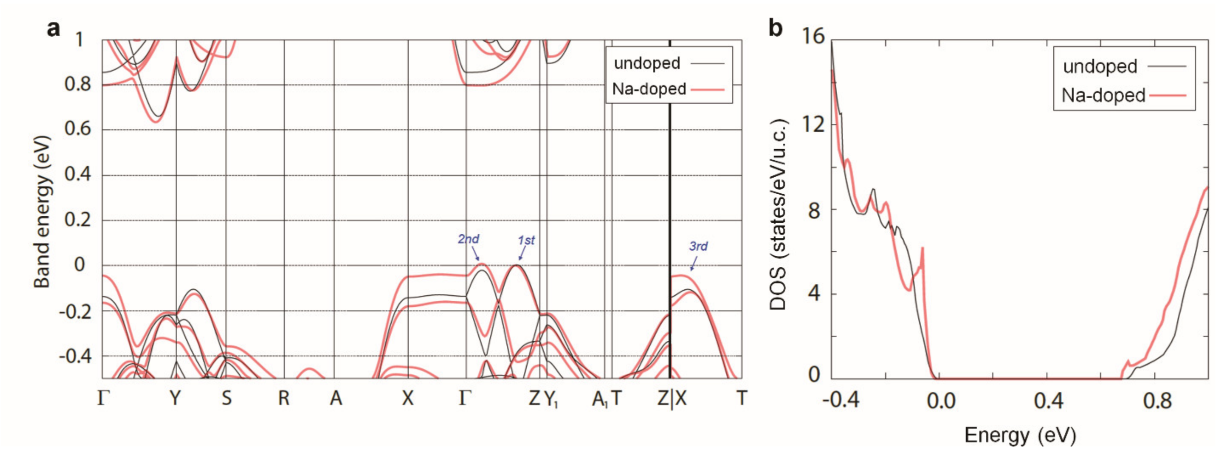

Supplementary Fig. 11. Theoretical calculations for electronic band structures of undoped and 3% Na-doped SnSe (*Pnma*). a, Electronic band structures. Carrier-contributing hole-pockets are indicated by 1st, 2nd, and 3rd. **b,** Density of states (DOS) low temperature *Pnma* phase based on DFT calculations. The electronic structures based on DFT calculations for undoped and 3% Na-doped SnSe of the low temperature *Pnma* phase show that Na-doping multiply modulates valence bands of SnSe as shown in **a**. First, the energy of a second valence band maximum (VBM_2) along the Γ-Z direction increases, making an energy gap between a first VBM (VBM_1) and VBM_2 negligible. Second, two doubly degenerated flat bands along the X-Γ and the X-direction split, and their upper energy band shifts to VBM_1. Consequently, carrier-contributing hole-pockets converge at the valence top level, resulting in the peak of density of states near the VBM as shown in **b**. This significantly improves *S* of Na-doped SnSe.

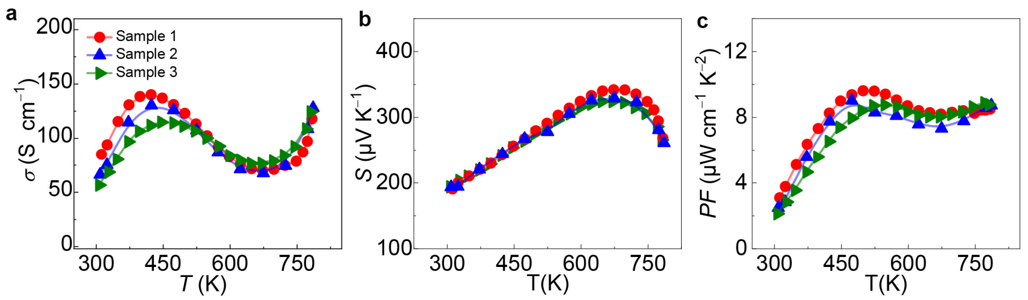

Supplementary Fig. 12. The reproducibility of thermoelectric properties as a function of temperature for the purified $Na_{0.03}Sn_{0.965}Se$. a, Electrical conductivity, *σ*. **b,** Seebeck coefficient, *S*. **c,** Power factor, *PF*. Data were measured along the SPS direction. The samples were independently prepared and measured.

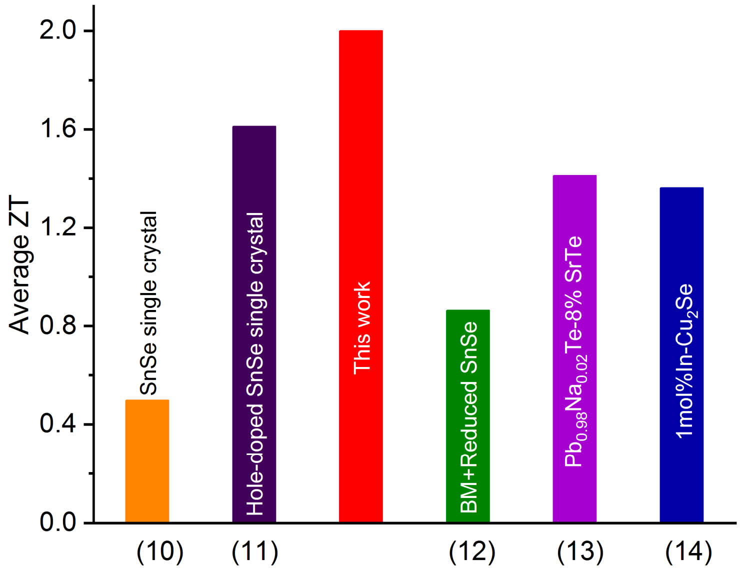

Supplementary Fig. 13. The average ZT of the purified polycrystalline $Na_{0.03}Sn_{0.965}Se$ sample between ~400 and ~780 K. The corresponding values for state-of the-art thermoelectric systems such as undoped SnSe single crystal[10], hole-doped SnSe single crystal[11], ball-milled and reduced $Na_{0.01}(Sn_{0.95}Pb_{0.05})_{0.99}Se$[12], $Pb_{0.98}Na_{0.02}Te$-8% SrTe[13], and 1 mol% In-Cu_2Se[14] are given for comparison. Unless noted as single crystal, all samples are polycrystalline. Because the cold side temperature T_c is closer to 400 K rather than 300 K in most practical thermoelectric devices for power generation, average ZT was calculated and compared in the temperature range between ~400 and ~780 K. We conservatively calculated the average ZT from various independent $Na_{0.03}Sn_{0.965}Se$ samples.

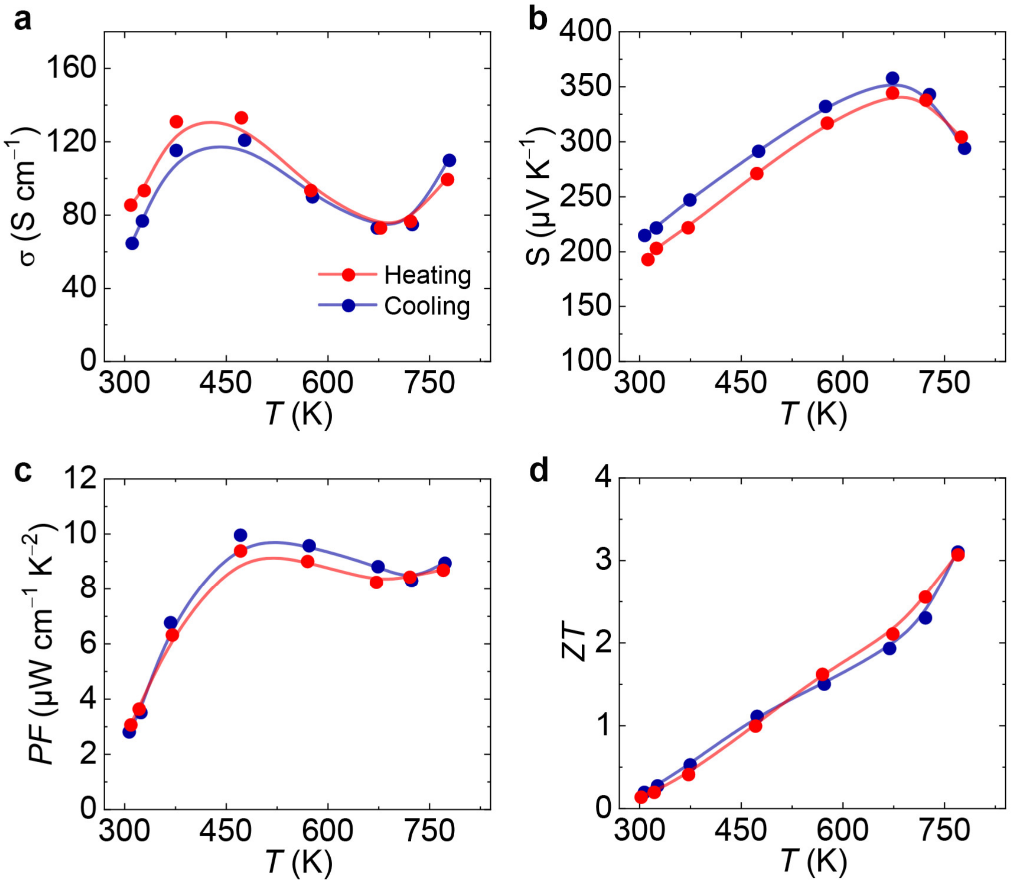

Supplementary Fig. 14. Temperature-dependent thermoelectric properties of the purified $Na_{0.03}Sn_{0.965}Se$ sample during the heating and cooling processes. a, Electrical conductivity, *σ*. **b,** Seebeck coefficient, *S*. **c,** Power factor, *PF*. **d,** thermoelectric figure of merit ZT.

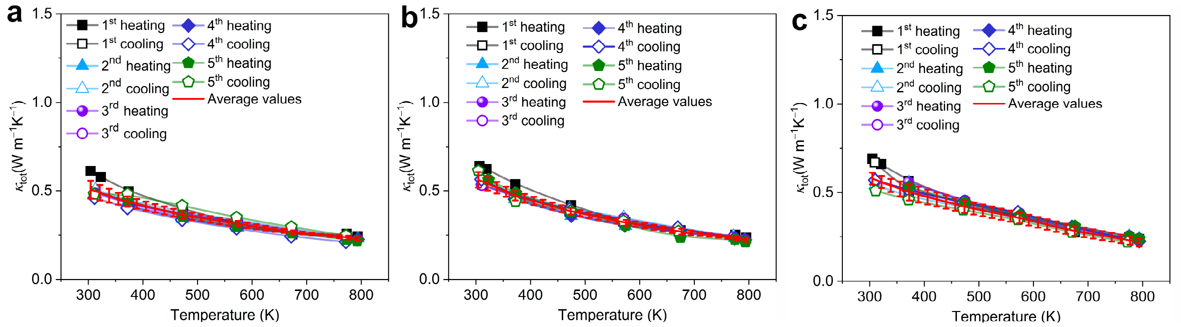

Supplementary Fig. 15. Temperature-dependent thermal conductivity of the purified $Na_{0.03}Sn_{0.965}Se$ samples for the five consecutive heating and cooling cycles. There samples were obtained from the independent synthesis batches. The average values are plotted in the red solid line with the experimental error bars according to the standard deviation of the data for the five round-trip cycles.

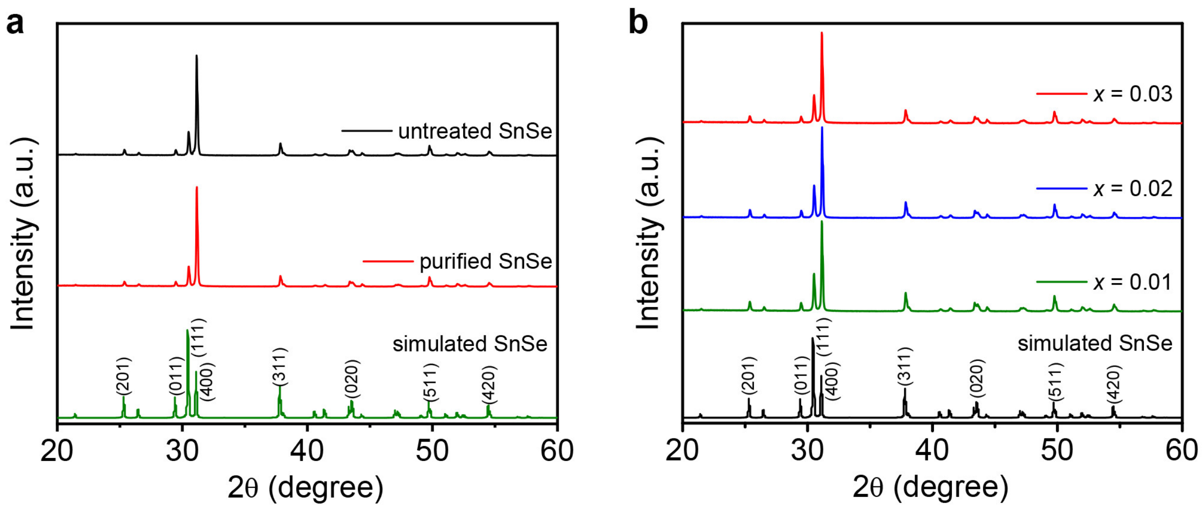

Supplementary Fig. 16. Powder X-ray diffraction (XRD) patterns. a, The untreated and purified SnSe samples. **b,** The purified $Na_xSn_{0.995-x}Se$ samples. The theoretically calculated pattern for SnSe (The International Centre for Diffraction Data (ICDD) PDF 01-075-6133) is given for comparison in **a** and **b**. Major Bragg peaks are indexed. SPS-processed ingots were ground to fine powders for the measurement.

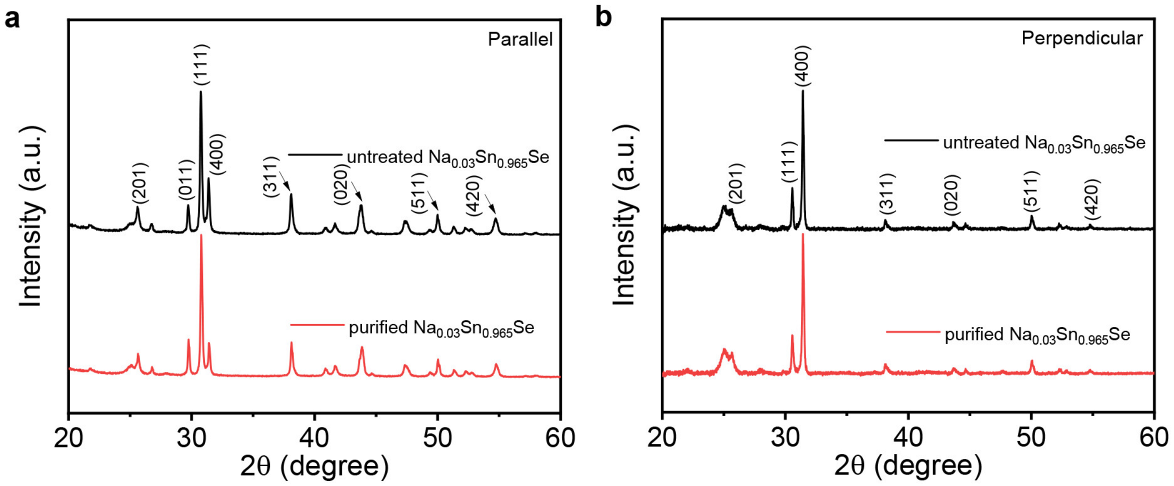

Supplementary Fig. 17. Powder XRD patterns measured **a** parallel and **b** perpendicular to the pressing direction of the SPS-processed ingots for purified and untreated $Na_{0.03}Sn_{0.965}Se$ samples. The Lotgering factor of the (111) with respect to the (*hkl*) reflections is 0.140 and 0.131 for the purified and untreated $Na_{0.03}Sn_{0.965}Se$ samples along the parallel direction to the SPS press, respectively. The results show that the degree of the preferred orientation is similar for the purified and untreated $Na_{0.03}Sn_{0.965}Se$ samples.

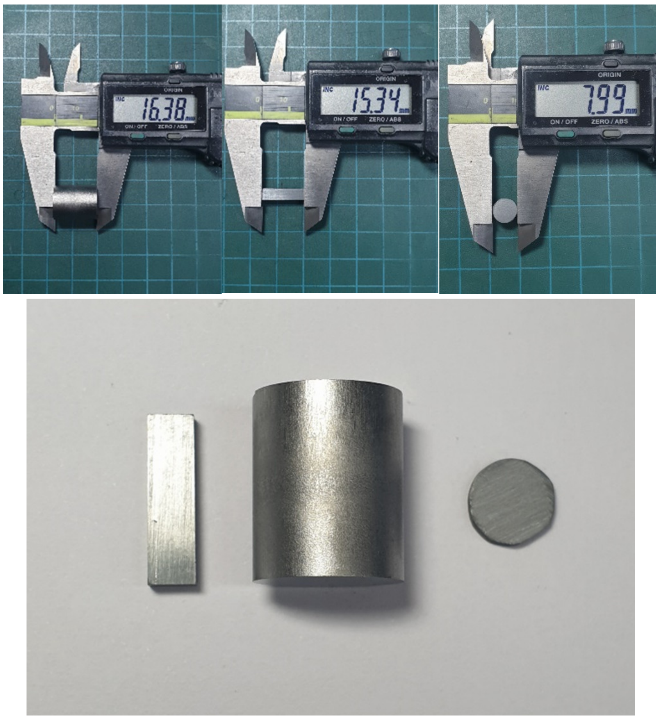

Supplementary Fig. 18. Typical dense pellets obtained by SPS with a diameter of 13 mm and a height of ~16 mm (center) and the specimens cut for measuring electrical (left, a bar with a length of ~15 mm) and thermal transport properties (right, a disk with a diameter of 8 mm) along the pressing direction of SPS. Their relative density is ~96%.

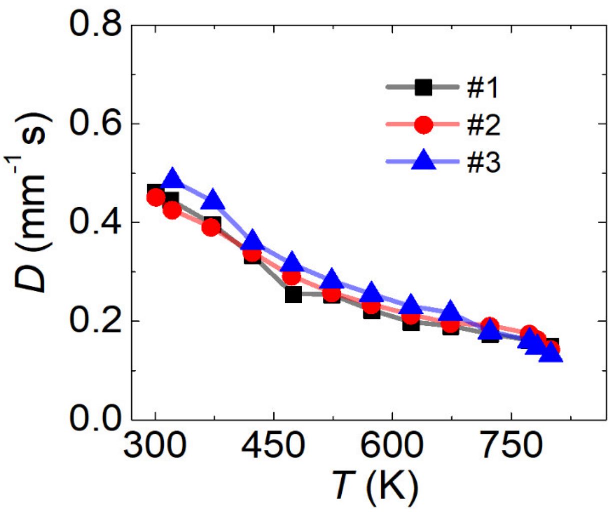

T (K)	#1 (mm^2·s^{-1})	#2 (mm^2·s^{-1})	#3 (mm^2·s^{-1})
300	0.462	0.451	-
323	0.445	0.425	0.485
373	0.396	0.390	0.442
423	0.333	0.339	0.360
473	0.254	0.291	0.315
523	0.253	0.257	0.281
573	0.221	0.233	0.254
623	0.197	0.212	0.229
673	0.189	0.195	0.216
723	0.173	0.190	0.178
773	0.160	0.174	0.160
783	0.156	0.163	0.146
800	0.148	0.143	0.131

Supplementary Fig. 19. Test results for thermal diffusivity from Northwestern University. 3 specimens have been prepared independently and measured simultaneously.

Supplementary Fig. 20. Test report for thermal diffusivity from NETZSCH Scientific Instruments Trading (Korea) Ltd. 3 specimens have been prepared independently and measured simultaneously.

Thermal Diffusivity - NETZSCH LFA Analysis

General information

**Database :**	nkslab-20012.mdb	**Remark(mment) :**	---
**Instrument :**	LFA 467	**Cp table :**	Cp_const
**Identity :**	NKSLAB-20012	**Expansion table :**	dL_const
**Date :**	2019-07-24	**Furnace :**	LFA 467 Steel
**Material :**	SNSE-1 - A1	**Sample holder :**	4 samples round/12.7mm
**Ref. density (20.0 °C) /(g/cm^3) :**	1.000	**Lamp :**	LFA 467 Flash Lamp
**Sample :**	SNSE-1	**Furnace TC :**	E
**Type :**	Single layer	**Sample TC :**	E
**Sample position :**	A1	**Sample Xp / Tn :**	4.00 / 4.00
**Detection Area (Diameter)/mm :**	4.0	**Furnace Xp / Tn :**	4.00 / 4.00
**Filter/% :**	0	**Calculation code :**	Standard + p.c./l/0-0-0
**Thickness (RT) /mm :**	1.6700	**Purge 1 MFC**	NITROGEN
**Diameter /mm :**	12.500	**Purge 2 MFC**	NITROGEN
**Sensor :**	InSb	**Protective MFC**	NITROGEN
**Operator :**	---		

Results

Shot number	Temperature °C	Model	Diffusivity mm^2/s	Uncertainty %	Laser voltage V	Pulse width ms
1	25.0	Standard + p.c.(l)	0.390	0.1	250.0	0.60
2	25.0	Standard + p.c.(l)	0.391	0.1	250.0	0.60
3	25.0	Standard + p.c.(l)	0.391	0.1	250.0	0.60
4	25.0	Standard + p.c.(l)	0.391	0.1	250.0	0.60
**Mean:**	**25.0**		**0.391**			
**Std. Dev.:**	**0.0**		**0.000**			
5	50.0	Standard + p.c.(l)	0.366	0.1	250.0	0.60
6	50.0	Standard + p.c.(l)	0.367	0.1	250.0	0.60
7	50.0	Standard + p.c.(l)	0.368	0.1	250.0	0.60
8	50.0	Standard + p.c.(l)	0.367	0.1	250.0	0.60
**Mean:**	**50.0**		**0.367**			
**Std. Dev.:**	**0.0**		**0.001**			

Created with NETZSCH Proteus software

Seonghwan Min, President
NETZSCH KOREA CO., LTD.
Odo-ro 15, Paju, Korea

Thermal Diffusivity - NETZSCH LFA Analysis

General information

**Database :**	nkslab-20012.mdb	**Remark(mment) :**	---
**Instrument :**	LFA 467	**Cp table :**	Cp_const
**Identity :**	NKSLAB-20012	**Expansion table :**	dL_const
**Date :**	2019-07-24	**Furnace :**	LFA 467 Steel
**Material :**	SNSE-1 - A1	**Sample holder :**	4 samples round/12.7mm
**Ref. density (20.0 °C) /(g/cm^3) :**	1.000	**Lamp :**	LFA 467 Flash Lamp
**Sample :**	SNSE-1	**Furnace TC :**	E
**Type :**	Single layer	**Sample TC :**	E
**Sample position :**	A1	**Sample Xp / Tn :**	4.00 / 4.00
**Detection Area (Diameter)/mm :**	4.0	**Furnace Xp / Tn :**	4.00 / 4.00
**Filter/% :**	0	**Calculation code :**	Standard + p.c./I/0-0-0
**Thickness (RT) /mm :**	1.6700	**Purge 1 MFC**	NITROGEN
**Diameter /mm :**	12.500	**Purge 2 MFC**	NITROGEN
**Sensor :**	InSb	**Protective MFC**	NITROGEN
**Operator :**	---		

Results

Shot number	Temperature °C	Model	Diffusivity mm^2/s	Uncertainty %	Laser voltage V	Pulse width ms
1	100.0	Standard + p.c.(I)	0.327	0.1	250.0	0.60
2	100.0	Standard + p.c.(I)	0.327	0.1	250.0	0.60
3	100.0	Standard + p.c.(I)	0.327	0.1	250.0	0.60
4	100.0	Standard + p.c.(I)	0.328	0.1	250.0	0.60
**Mean:**	**100.0**		**0.327**			
**Std. Dev.:**	**0.0**		**0.001**			

Created with NETZSCH Proteus software

Seongnwan Min, President
NETZSCH KOREA CO., LTD.
Odo-ro 15, Paju, Korea

Thermal Diffusivity - NETZSCH LFA Analysis

General information

**Database :**	nkslab-20012.mdb	**Remark(mment) :**	---
**Instrument :**	LFA 467	**Cp table :**	Cp_const
**Identity :**	NKSLAB-20012	**Expansion table :**	dL_const
**Date :**	2019-07-24	**Furnace :**	LFA 467 Steel
**Material :**	SNSE-1 - A1	**Sample holder :**	4 samples round/12.7mm
**Ref. density (20.0 °C) /(g/cm^3) :**	1.000	**Lamp :**	LFA 467 Flash Lamp
**Sample :**	SNSE-1	**Furnace TC :**	E
**Type :**	Single layer	**Sample TC :**	E
**Sample position :**	A1	**Sample Xp / Tn :**	4.00 / 4.00
**Detection Area (Diameter)/mm :**	4.0	**Furnace Xp / Tn :**	4.00 / 4.00
**Filter/% :**	0	**Calculation code :**	Standard + p.c./I/0-0-0
**Thickness (RT) /mm :**	1.6700	**Purge 1 MFC**	NITROGEN
**Diameter /mm :**	12.500	**Purge 2 MFC**	NITROGEN
**Sensor :**	InSb	**Protective MFC**	NITROGEN
**Operator :**	---		

Results

Shot number	Temperature °C	Model	Diffusivity mm^2/s	Uncertainty %	Laser voltage V	Pulse width ms
1	150.0	Standard + p.c.(I)	0.292	0.1	250.0	0.60
2	150.0	Standard + p.c.(I)	0.293	0.1	250.0	0.60
3	150.0	Standard + p.c.(I)	0.293	0.1	250.0	0.60
4	150.0	Standard + p.c.(I)	0.293	0.1	250.0	0.60
**Mean:**	**150.0**		**0.293**			
**Std. Dev.:**	**0.0**		**0.000**			
5	200.0	Standard + p.c.(I)	0.260	0.1	250.0	0.60
6	200.0	Standard + p.c.(I)	0.260	0.1	250.0	0.60
7	200.0	Standard + p.c.(I)	0.259	0.1	250.0	0.60
8	200.0	Standard + p.c.(I)	0.258	0.1	250.0	0.60
**Mean:**	**200.0**		**0.259**			
**Std. Dev.:**	**0.0**		**0.001**			
9	250.0	Standard + p.c.(I)	0.231	0.1	250.0	0.60
10	250.0	Standard + p.c.(I)	0.232	0.1	250.0	0.60
11	250.0	Standard + p.c.(I)	0.232	0.1	250.0	0.60
12	250.0	Standard + p.c.(I)	0.232	0.1	250.0	0.60
**Mean:**	**250.0**		**0.232**			
**Std. Dev.:**	**0.0**		**0.000**			
13	300.0	Standard + p.c.(I)	0.211	0.1	250.0	0.60
14	300.0	Standard + p.c.(I)	0.212	0.1	250.0	0.60
15	300.0	Standard + p.c.(I)	0.212	0.1	250.0	0.60
16	300.0	Standard + p.c.(I)	0.209	0.2	250.0	0.60
**Mean:**	**300.0**		**0.211**			
**Std. Dev.:**	**0.0**		**0.002**			
17	350.0	Standard + p.c.(I)	0.193	0.1	250.0	0.60
18	350.0	Standard + p.c.(I)	0.196	0.2	250.0	0.60
19	350.0	Standard + p.c.(I)	0.191	0.1	250.0	0.60
20	350.0	Standard + p.c.(I)	0.190	0.1	250.0	0.60
**Mean:**	**350.0**		**0.192**			
**Std. Dev.:**	**0.0**		**0.003**			
21	400.0	Standard + p.c.(I)	0.173	0.1	250.0	0.60
22	400.0	Standard + p.c.(I)	0.172	0.1	250.0	0.60
23	400.0	Standard + p.c.(I)	0.171	0.2	250.0	0.60
24	400.0	Standard + p.c.(I)	0.170	0.1	250.0	0.60
**Mean:**	**400.0**		**0.172**			
**Std. Dev.:**	**0.0**		**0.001**			
25	450.0	Standard + p.c.(I)	0.161	0.1	250.0	0.60
26	450.0	Standard + p.c.(I)	0.160	0.1	250.0	0.60
27	450.0	Standard + p.c.(I)	0.159	0.1	250.0	0.60
28	450.0	Standard + p.c.(I)	0.158	0.1	250.0	0.60
29	450.0	Standard + p.c.(I)	0.158	0.2	250.0	0.60
**Mean:**	**450.0**		**0.159**			
**Std. Dev.:**	**0.0**		**0.001**			

Created with NETZSCH Proteus software

Seonghwan Min, President
NETZSCH KOREA CO., LTD.
Odo-ro 15, Paju, Korea

Thermal Diffusivity - NETZSCH LFA Analysis

General information

**Database :**	nkslab-20012.mdb	**Remark(mment) :**	---
**Instrument :**	LFA 467	**Cp table :**	Cp_const
**Identity :**	NKSLAB-20012	**Expansion table :**	dL_const
**Date :**	2019-07-24	**Furnace :**	LFA 467 Steel
**Material :**	SNSE-1 - A1	**Sample holder :**	4 samples round/12.7mm
**Ref. density (20.0 °C) /(g/cm^3) :**	1.000	**Lamp :**	LFA 467 Flash Lamp
**Sample :**	SNSE-1	**Furnace TC :**	E
**Type :**	Single layer	**Sample TC :**	E
**Sample position :**	A1	**Sample Xp / Tn :**	4.00 / 4.00
**Detection Area (Diameter)/mm :**	4.0	**Furnace Xp / Tn :**	4.00 / 4.00
**Filter/% :**	0	**Calculation code :**	Standard + p.c./l/0-0-0
**Thickness (RT) /mm :**	1.6700	**Purge 1 MFC**	NITROGEN
**Diameter /mm :**	12.500	**Purge 2 MFC**	NITROGEN
**Sensor :**	InSb	**Protective MFC**	NITROGEN
**Operator :**	---		

Results

Shot number	Temperature °C	Model	Diffusivity mm^2/s	Uncertainty %	Laser voltage V	Pulse width ms
1	500.0	Standard + p.c.(l)	0.157	0.3	250.0	0.60
2	500.0	Standard + p.c.(l)	0.150	0.1	250.0	0.60
3	500.0	Standard + p.c.(l)	0.148	0.1	250.0	0.60
4	500.0	Standard + p.c.(l)	0.149	0.1	250.0	0.60
5	500.0	Standard + p.c.(l)	0.148	0.1	250.0	0.60
**Mean:**	**500.0**		**0.150**			
**Std. Dev.:**	**0.0**		**0.004**			

Created with NETZSCH Proteus software

Seonghwan Min, President
NETZSCH KOREA CO., LTD.
Odo-ro 15, Paju, Korea

Thermal Diffusivity - NETZSCH LFA Analysis

General information

**Database :**	nkslab-20012.mdb	**Remark(mment) :**	---
**Instrument :**	LFA 467	**Cp table :**	Cp_const
**Identity :**	NKSLAB-20012	**Expansion table :**	dL_const
**Date :**	2019-07-24	**Furnace :**	LFA 467 Steel
**Material :**	SNSE-2 - A2	**Sample holder :**	4 samples round/12.7mm
**Ref. density (20.0 °C) /(g/cm^3) :**	1.000	**Lamp :**	LFA 467 Flash Lamp
**Sample :**	SNSE-2	**Furnace TC :**	E
**Type :**	Single layer	**Sample TC :**	E
**Sample position :**	A2	**Sample Xp / Tn :**	4.00 / 4.00
**Detection Area (Diameter)/mm :**	4.0	**Furnace Xp / Tn :**	4.00 / 4.00
**Filter/% :**	0	**Calculation code :**	Standard + p.c./I/0-0-0
**Thickness (RT) /mm :**	1.6400	**Purge 1 MFC**	NITROGEN
**Diameter /mm :**	12.500	**Purge 2 MFC**	NITROGEN
**Sensor :**	InSb	**Protective MFC**	NITROGEN
**Operator :**	---		

Results

Shot number	Temperature °C	Model	Diffusivity mm^2/s	Uncertainty %	Laser voltage V	Pulse width ms
1	25.0	Standard + p.c.(I)	0.442	0.1	250.0	0.60
2	25.0	Standard + p.c.(I)	0.442	0.1	250.0	0.60
3	25.0	Standard + p.c.(I)	0.443	0.1	250.0	0.60
4	25.0	Standard + p.c.(I)	0.443	0.1	250.0	0.60
**Mean:**	**25.0**		**0.443**			
**Std. Dev.:**	**0.0**		**0.000**			
5	50.0	Standard + p.c.(I)	0.414	0.1	250.0	0.60
6	50.0	Standard + p.c.(I)	0.414	0.1	250.0	0.60
7	50.0	Standard + p.c.(I)	0.412	0.1	250.0	0.60
8	50.0	Standard + p.c.(I)	0.413	0.1	250.0	0.60
**Mean:**	**50.0**		**0.413**			
**Std. Dev.:**	**0.0**		**0.001**			

Created with NETZSCH Proteus software

Seonghwan Min, President
NETZSCH KOREA CO., LTD.
Odo-ro 15, Paju, Korea

Thermal Diffusivity - NETZSCH LFA Analysis

General information

**Database :**	nkslab-20012.mdb	**Remark(mment) :**	---
**Instrument :**	LFA 467	**Cp table :**	Cp_const
**Identity :**	NKSLAB-20012	**Expansion table :**	dL_const
**Date :**	2019-07-24	**Furnace :**	LFA 467 Steel
**Material :**	SNSE-2 - A2	**Sample holder :**	4 samples round/12.7mm
**Ref. density (20.0 °C) /(g/cm^3) :**	1.000	**Lamp :**	LFA 467 Flash Lamp
**Sample :**	SNSE-2	**Furnace TC :**	E
**Type :**	Single layer	**Sample TC :**	E
**Sample position :**	A2	**Sample Xp / Tn :**	4.00 / 4.00
**Detection Area (Diameter)/mm :**	4.0	**Furnace Xp / Tn :**	4.00 / 4.00
**Filter/% :**	0	**Calculation code :**	Standard + p.c./l/0-0-0
**Thickness (RT) /mm :**	1.6400	**Purge 1 MFC**	NITROGEN
**Diameter /mm :**	12.500	**Purge 2 MFC**	NITROGEN
**Sensor :**	InSb	**Protective MFC**	NITROGEN
**Operator :**	---		

Results

Shot number	Temperature °C	Model	Diffusivity mm^2/s	Uncertainty %	Laser voltage V	Pulse width ms
1	100.0	Standard + p.c.(l)	0.365	0.1	250.0	0.60
2	100.0	Standard + p.c.(l)	0.366	0.1	250.0	0.60
3	100.0	Standard + p.c.(l)	0.365	0.1	250.0	0.60
4	100.0	Standard + p.c.(l)	0.366	0.1	250.0	0.60
**Mean:**	**100.0**		**0.365**			
**Std. Dev.:**	**0.0**		**0.000**			

Created with NETZSCH Proteus software

Seonghwan Min, President
NETZSCH KOREA CO., LTD.
Odo-ro 15, Paju, Korea

Thermal Diffusivity - NETZSCH LFA Analysis

General information

**Database :**	nkslab-20012.mdb	**Remark(mment) :**	---
**Instrument :**	LFA 467	**Cp table :**	Cp_const
**Identity :**	NKSLAB-20012	**Expansion table :**	dL_const
**Date :**	2019-07-24	**Furnace :**	LFA 467 Steel
**Material :**	SNSE-2 - A2	**Sample holder :**	4 samples round/12.7mm
**Ref. density (20.0 °C) /(g/cm^3) :**	1.000	**Lamp :**	LFA 467 Flash Lamp
**Sample :**	SNSE-2	**Furnace TC :**	E
**Type :**	Single layer	**Sample TC :**	E
**Sample position :**	A2	**Sample Xp / Tn :**	4.00 / 4.00
**Detection Area (Diameter)/mm :**	4.0	**Furnace Xp / Tn :**	4.00 / 4.00
**Filter/% :**	0	**Calculation code :**	Standard + p.c./l/0-0-0
**Thickness (RT) /mm :**	1.6400	**Purge 1 MFC**	NITROGEN
**Diameter /mm :**	12.500	**Purge 2 MFC**	NITROGEN
**Sensor :**	InSb	**Protective MFC**	NITROGEN
**Operator :**	---		

Results

Shot number	Temperature °C	Model	Diffusivity mm^2/s	Uncertainty %	Laser voltage V	Pulse width ms
1	150.0	Standard + p.c.(l)	0.325	0.1	250.0	0.60
2	150.0	Standard + p.c.(l)	0.325	0.1	250.0	0.60
3	150.0	Standard + p.c.(l)	0.325	0.1	250.0	0.60
4	150.0	Standard + p.c.(l)	0.325	0.1	250.0	0.60
**Mean:**	**150.0**		**0.325**			
**Std. Dev.:**	**0.0**		**0.000**			
5	200.0	Standard + p.c.(l)	0.288	0.1	250.0	0.60
6	200.0	Standard + p.c.(l)	0.287	0.1	250.0	0.60
7	200.0	Standard + p.c.(l)	0.287	0.1	250.0	0.60
8	200.0	Standard + p.c.(l)	0.287	0.1	250.0	0.60
**Mean:**	**200.0**		**0.287**			
**Std. Dev.:**	**0.0**		**0.001**			
9	250.0	Standard + p.c.(l)	0.256	0.1	250.0	0.60
10	250.0	Standard + p.c.(l)	0.257	0.1	250.0	0.60
11	250.0	Standard + p.c.(l)	0.257	0.1	250.0	0.60
12	250.0	Standard + p.c.(l)	0.257	0.1	250.0	0.60
**Mean:**	**250.0**		**0.257**			
**Std. Dev.:**	**0.0**		**0.000**			
13	300.0	Standard + p.c.(l)	0.233	0.1	250.0	0.60
14	300.0	Standard + p.c.(l)	0.234	0.1	250.0	0.60
15	300.0	Standard + p.c.(l)	0.233	0.1	250.0	0.60
16	300.0	Standard + p.c.(l)	0.233	0.1	250.0	0.60
**Mean:**	**300.0**		**0.233**			
**Std. Dev.:**	**0.0**		**0.001**			
17	350.0	Standard + p.c.(l)	0.208	0.1	250.0	0.60
18	350.0	Standard + p.c.(l)	0.208	0.1	250.0	0.60
19	350.0	Standard + p.c.(l)	0.209	0.1	250.0	0.60
20	350.0	Standard + p.c.(l)	0.207	0.1	250.0	0.60
**Mean:**	**350.0**		**0.208**			
**Std. Dev.:**	**0.0**		**0.001**			
21	400.0	Standard + p.c.(l)	0.187	0.1	250.0	0.60
22	400.0	Standard + p.c.(l)	0.187	0.1	250.0	0.60
23	400.0	Standard + p.c.(l)	0.188	0.1	250.0	0.60
24	400.0	Standard + p.c.(l)	0.187	0.1	250.0	0.60
**Mean:**	**400.0**		**0.187**			
**Std. Dev.:**	**0.0**		**0.000**			
25	450.0	Standard + p.c.(l)	0.175	0.2	250.0	0.60
26	450.0	Standard + p.c.(l)	0.171	0.1	250.0	0.60
27	450.0	Standard + p.c.(l)	0.171	0.1	250.0	0.60
28	450.0	Standard + p.c.(l)	0.172	0.2	250.0	0.60
29	450.0	Standard + p.c.(l)	0.172	0.1	250.0	0.60
**Mean:**	**450.0**		**0.172**			
**Std. Dev.:**	**0.0**		**0.002**			

Created with NETZSCH Proteus software

Seonghwan Min, President
NETZSCH KOREA CO., LTD.
Odo-ro 15, Paju, Korea

Thermal Diffusivity - NETZSCH LFA Analysis

General information

**Database :**	nkslab-20012.mdb	**Remark(mment) :**	---
**Instrument :**	LFA 467	**Cp table :**	Cp_const
**Identity :**	NKSLAB-20012	**Expansion table :**	dL_const
**Date :**	2019-07-24	**Furnace :**	LFA 467 Steel
**Material :**	SNSE-2 - A2	**Sample holder :**	4 samples round/12.7mm
**Ref. density (20.0 °C) /(g/cm^3) :**	1.000	**Lamp :**	LFA 467 Flash Lamp
**Sample :**	SNSE-2	**Furnace TC :**	E
**Type :**	Single layer	**Sample TC :**	E
**Sample position :**	A2	**Sample Xp / Tn :**	4.00 / 4.00
**Detection Area (Diameter)/mm :**	4.0	**Furnace Xp / Tn :**	4.00 / 4.00
**Filter/% :**	0	**Calculation code :**	Standard + p.c./I/0-0-0
**Thickness (RT) /mm :**	1.6400	**Purge 1 MFC**	NITROGEN
**Diameter /mm :**	12.500	**Purge 2 MFC**	NITROGEN
**Sensor :**	InSb	**Protective MFC**	NITROGEN
**Operator :**	---		

Results

Shot number	Temperature °C	Model	Diffusivity mm^2/s	Uncertainty %	Laser voltage V	Pulse width ms
1	500.0	Standard + p.c.(I)	0.164	0.1	250.0	0.60
2	500.0	Standard + p.c.(I)	0.160	0.1	250.0	0.60
3	500.0	Standard + p.c.(I)	0.166	0.2	250.0	0.60
4	500.0	Standard + p.c.(I)	0.162	0.1	250.0	0.60
5	500.0	Standard + p.c.(I)	0.162	0.1	250.0	0.60
**Mean:**	**500.0**		**0.163**			
**Std. Dev.:**	**0.0**		**0.002**			

Created with NETZSCH Proteus software

Seonghwan Min, President
NETZSCH KOREA CO., LTD.
Odo-ro 15, Paju, Korea

Thermal Diffusivity - NETZSCH LFA Analysis

General information

**Database :**	nkslab-20012.mdb	**Remark(mment) :**	---
**Instrument :**	LFA 467	**Cp table :**	Cp_const
**Identity :**	NKSLAB-20012	**Expansion table :**	dL_const
**Date :**	2019-07-24	**Furnace :**	LFA 467 Steel
**Material :**	SNSE-3 - A3	**Sample holder :**	4 samples round/12.7mm
**Ref. density (20.0 °C) /(g/cm^3) :**	1.000	**Lamp :**	LFA 467 Flash Lamp
**Sample :**	SNSE-3	**Furnace TC :**	E
**Type :**	Single layer	**Sample TC :**	E
**Sample position :**	A3	**Sample Xp / Tn :**	4.00 / 4.00
**Detection Area (Diameter)/mm :**	4.0	**Furnace Xp / Tn :**	4.00 / 4.00
**Filter/% :**	0	**Calculation code :**	Standard + p.c./I/0-0-0
**Thickness (RT) /mm :**	1.5800	**Purge 1 MFC**	NITROGEN
**Diameter /mm :**	12.500	**Purge 2 MFC**	NITROGEN
**Sensor :**	InSb	**Protective MFC**	NITROGEN
**Operator :**	---		

Results

Shot number	Temperature °C	Model	Diffusivity mm^2/s	Uncertainty %	Laser voltage V	Pulse width ms
1	25.0	Standard + p.c.(I)	0.395	0.1	250.0	0.60
2	25.0	Standard + p.c.(I)	0.395	0.1	250.0	0.60
3	25.0	Standard + p.c.(I)	0.394	0.1	250.0	0.60
4	25.0	Standard + p.c.(I)	0.396	0.1	250.0	0.60
**Mean:**	**25.0**		**0.395**			
**Std. Dev.:**	**0.0**		**0.001**			
5	50.0	Standard + p.c.(I)	0.369	0.1	250.0	0.60
6	50.0	Standard + p.c.(I)	0.370	0.1	250.0	0.60
7	50.0	Standard + p.c.(I)	0.369	0.1	250.0	0.60
8	50.0	Standard + p.c.(I)	0.370	0.1	250.0	0.60
**Mean:**	**50.0**		**0.369**			
**Std. Dev.:**	**0.0**		**0.000**			

Created with NETZSCH Proteus software

Seonghwan Min, President
NETZSCH KOREA CO., LTD.
Odo-ro 15, Paju, Korea

Thermal Diffusivity - NETZSCH LFA Analysis

General information

**Database :**	nkslab-20012.mdb	**Remark(mment) :**	---
**Instrument :**	LFA 467	**Cp table :**	Cp_const
**Identity :**	NKSLAB-20012	**Expansion table :**	dL_const
**Date :**	2019-07-24	**Furnace :**	LFA 467 Steel
**Material :**	SNSE-3 - A3	**Sample holder :**	4 samples round/12.7mm
**Ref. density (20.0 °C) /(g/cm^3) :**	1.000	**Lamp :**	LFA 467 Flash Lamp
**Sample :**	SNSE-3	**Furnace TC :**	E
**Type :**	Single layer	**Sample TC :**	E
**Sample position :**	A3	**Sample Xp / Tn :**	4.00 / 4.00
**Detection Area (Diameter)/mm :**	4.0	**Furnace Xp / Tn :**	4.00 / 4.00
**Filter/% :**	0	**Calculation code :**	Standard + p.c./I/0-0-0
**Thickness (RT) /mm :**	1.5800	**Purge 1 MFC**	NITROGEN
**Diameter /mm :**	12.500	**Purge 2 MFC**	NITROGEN
**Sensor :**	InSb	**Protective MFC**	NITROGEN
**Operator :**	---		

Results

Shot number	Temperature °C	Model	Diffusivity mm^2/s	Uncertainty %	Laser voltage V	Pulse width ms
1	100.0	Standard + p.c.(l)	0.328	0.1	250.0	0.60
2	100.0	Standard + p.c.(l)	0.328	0.1	250.0	0.60
3	100.0	Standard + p.c.(l)	0.329	0.1	250.0	0.60
4	100.0	Standard + p.c.(l)	0.329	0.1	250.0	0.60
**Mean:**	**100.0**		**0.329**			
**Std. Dev.:**	**0.0**		**0.000**			

Created with NETZSCH Proteus software

Seonghwan Min, President
NETZSCH KOREA CO., LTD.
Odo-ro 15, Paju, Korea

Thermal Diffusivity - NETZSCH LFA Analysis

General information

**Database :**	nkslab-20012.mdb	**Remark(mment) :**	---
**Instrument :**	LFA 467	**Cp table :**	Cp_const
**Identity :**	NKSLAB-20012	**Expansion table :**	dL_const
**Date :**	2019-07-24	**Furnace :**	LFA 467 Steel
**Material :**	SNSE-3 - A3	**Sample holder :**	4 samples round/12.7mm
**Ref. density (20.0 °C) /(g/cm^3) :**	1.000	**Lamp :**	LFA 467 Flash Lamp
**Sample :**	SNSE-3	**Furnace TC :**	E
**Type :**	Single layer	**Sample TC :**	E
**Sample position :**	A3	**Sample Xp / Tn :**	4.00 / 4.00
**Detection Area (Diameter)/mm :**	4.0	**Furnace Xp / Tn :**	4.00 / 4.00
**Filter/% :**	0	**Calculation code :**	Standard + p.c./I/0-0-0
**Thickness (RT) /mm :**	1.5800	**Purge 1 MFC**	NITROGEN
**Diameter /mm :**	12.500	**Purge 2 MFC**	NITROGEN
**Sensor :**	InSb	**Protective MFC**	NITROGEN
**Operator :**	---		

Results

Shot number	Temperature °C	Model	Diffusivity mm^2/s	Uncertainty %	Laser voltage V	Pulse width ms
1	150.0	Standard + p.c.(l)	0.292	0.1	250.0	0.60
2	150.0	Standard + p.c.(l)	0.293	0.1	250.0	0.60
3	150.0	Standard + p.c.(l)	0.293	0.1	250.0	0.60
4	150.0	Standard + p.c.(l)	0.292	0.1	250.0	0.60
**Mean:**	**150.0**		**0.293**			
**Std. Dev.:**	**0.0**		**0.000**			
5	200.0	Standard + p.c.(l)	0.255	0.1	250.0	0.60
6	200.0	Standard + p.c.(l)	0.255	0.1	250.0	0.60
7	200.0	Standard + p.c.(l)	0.255	0.1	250.0	0.60
8	200.0	Standard + p.c.(l)	0.254	0.1	250.0	0.60
**Mean:**	**200.0**		**0.255**			
**Std. Dev.:**	**0.0**		**0.000**			
9	250.0	Standard + p.c.(l)	0.230	0.1	250.0	0.60
10	250.0	Standard + p.c.(l)	0.231	0.1	250.0	0.60
11	250.0	Standard + p.c.(l)	0.231	0.1	250.0	0.60
12	250.0	Standard + p.c.(l)	0.231	0.1	250.0	0.60
**Mean:**	**250.0**		**0.231**			
**Std. Dev.:**	**0.0**		**0.000**			
13	300.0	Standard + p.c.(l)	0.207	0.2	250.0	0.60
14	300.0	Standard + p.c.(l)	0.209	0.1	250.0	0.60
15	300.0	Standard + p.c.(l)	0.208	0.1	250.0	0.60
16	300.0	Standard + p.c.(l)	0.209	0.1	250.0	0.60
**Mean:**	**300.0**		**0.208**			
**Std. Dev.:**	**0.0**		**0.001**			
17	350.0	Standard + p.c.(l)	0.186	0.1	250.0	0.60
18	350.0	Standard + p.c.(l)	0.187	0.1	250.0	0.60
19	350.0	Standard + p.c.(l)	0.187	0.1	250.0	0.60
20	350.0	Standard + p.c.(l)	0.187	0.1	250.0	0.60
**Mean:**	**350.0**		**0.187**			
**Std. Dev.:**	**0.0**		**0.001**			
21	400.0	Standard + p.c.(l)	0.168	0.1	250.0	0.60
22	400.0	Standard + p.c.(l)	0.169	0.1	250.0	0.60
23	400.0	Standard + p.c.(l)	0.167	0.1	250.0	0.60
24	400.0	Standard + p.c.(l)	0.168	0.1	250.0	0.60
**Mean:**	**400.0**		**0.168**			
**Std. Dev.:**	**0.0**		**0.001**			
25	450.0	Standard + p.c.(l)	0.158	0.2	250.0	0.60
26	450.0	Standard + p.c.(l)	0.155	0.1	250.0	0.60
27	450.0	Standard + p.c.(l)	0.160	0.1	250.0	0.60
28	450.0	Standard + p.c.(l)	0.157	0.1	250.0	0.60
29	450.0	Standard + p.c.(l)	0.159	0.1	250.0	0.60
**Mean:**	**450.0**		**0.158**			
**Std. Dev.:**	**0.0**		**0.002**			

Created with NETZSCH Proteus software

Seonghwan Min, President
NETZSCH KOREA CO., LTD.
Odo-ro 15, Paju, Korea

Thermal Diffusivity - NETZSCH LFA Analysis

General information

**Database :**	nkslab-20012.mdb	**Remark(mment) :**	---
**Instrument :**	LFA 467	**Cp table :**	Cp_const
**Identity :**	NKSLAB-20012	**Expansion table :**	dL_const
**Date :**	2019-07-24	**Furnace :**	LFA 467 Steel
**Material :**	SNSE-3 - A3	**Sample holder :**	4 samples round/12.7mm
**Ref. density (20.0 °C) /(g/cm^3) :**	1.000	**Lamp :**	LFA 467 Flash Lamp
**Sample :**	SNSE-3	**Furnace TC :**	E
**Type :**	Single layer	**Sample TC :**	E
**Sample position :**	A3	**Sample Xp / Tn :**	4.00 / 4.00
**Detection Area (Diameter)/mm :**	4.0	**Furnace Xp / Tn :**	4.00 / 4.00
**Filter/% :**	0	**Calculation code :**	Standard + p.c./I/0-0-0
**Thickness (RT) /mm :**	1.5800	**Purge 1 MFC**	NITROGEN
**Diameter /mm :**	12.500	**Purge 2 MFC**	NITROGEN
**Sensor :**	InSb	**Protective MFC**	NITROGEN
**Operator :**	---		

Results

Shot number	Temperature °C	Model	Diffusivity mm^2/s	Uncertainty %	Laser voltage V	Pulse width ms
1	500.0	Standard + p.c.(I)	0.150	0.1	250.0	0.60
2	500.0	Standard + p.c.(I)	0.149	0.1	250.0	0.60
3	500.0	Standard + p.c.(I)	0.146	0.2	250.0	0.60
4	500.0	Standard + p.c.(I)	0.149	0.1	250.0	0.60
5	500.0	Standard + p.c.(I)	0.148	0.1	250.0	0.60
**Mean:**	**500.0**		**0.149**			
**Std. Dev.:**	**0.0**		**0.001**			

Created with NETZSCH Proteus software

Seonghwan Min, President
NETZSCH KOREA CO., LTD.
Odo-ro 15, Paju, Korea

Supplementary Fig. 21. Test report for thermal diffusivity from Seoul National University. 4 specimens have been prepared independently and measured simultaneously.

Thermal Diffusivity - NETZSCH LFA Analysis

General information

**Database :**	yklee.mdb	**Operator :**	YKLee
**Instrument :**	LFA 457	**Customer :**	YKLee
**Identity :**	SNU SnSe	**Remark(mment) :**	w/ graphite
**Date :**	12/23/2019	**Cp table :**	Cp_const
**Material :**	purified Na0.03Sn0.965Se	**Expansion table :**	undefined
**Ref. density (20.0 °C) /(g/cm^3) :**	5.910	**Temp. recalib. file :**	undefined
**Sample :**	purified Na0.03Sn0.965Se	**Purge gas :**	used
**Type :**	Single layer	**Furnace :**	LFA 457 Medium Rg
**Coating :**	yes	**Sample holder :**	unknown
**Thickness (RT) /mm :**	1.2400	**Laser :**	LFA 457 Laser
**Diameter /mm :**	6.000	**Centering cone :**	Std SiC 6.0mm
**Sensor :**	InSb	**Center cone ratio :**	0.77
**Beam enlargement : /mm**	12.7	**Furnace TC :**	S
**Laser filter : /%**	100.0	**Sample TC :**	S
**Atmosphere :**	Ar	**Sample Xp / Tn :**	2.40 / 0.60
**Gas flow : /(ml/min)**	100.00	**Furnace Xp / Tn :**	3.00 / 0.60
**Laboratory :**	EML	**Calculation code :**	C+p/l/0-x-0

Results

Shot number	Temperature °C	Model	Diffusivity mm^2/s	Laser voltage V
1	29.5	Cowan + pc.	0.462	1730.0
2	29.3	Cowan + pc.	0.461	1730.0
3	29.1	Cowan + pc.	0.436	1730.0
**Mean:**	**29.3**		**0.453**	
**Std. Dev.:**	**0.2**		**0.015**	
4	49.3	Cowan + pc.	0.430	1730.0
5	49.3	Cowan + pc.	0.419	1730.0
6	49.7	Cowan + pc.	0.439	1730.0
**Mean:**	**49.4**		**0.429**	
**Std. Dev.:**	**0.2**		**0.010**	
7	101.0	Cowan + pc.	0.361	1730.0
8	100.8	Cowan + pc.	0.359	1730.0
9	100.5	Cowan + pc.	0.360	1730.0
**Mean:**	**100.8**		**0.360**	
**Std. Dev.:**	**0.3**		**0.001**	
10	150.5	Cowan + pc.	0.313	1730.0
11	150.4	Cowan + pc.	0.312	1730.0
12	150.3	Cowan + pc.	0.318	1730.0
**Mean:**	**150.4**		**0.314**	
**Std. Dev.:**	**0.1**		**0.003**	
13	200.4	Cowan + pc.	0.271	1730.0
14	200.3	Cowan + pc.	0.275	1730.0
15	200.2	Cowan + pc.	0.271	1730.0
**Mean:**	**200.3**		**0.272**	
**Std. Dev.:**	**0.1**		**0.002**	
16	250.4	Cowan + pc.	0.238	1730.0
17	250.3	Cowan + pc.	0.230	1730.0
18	250.2	Cowan + pc.	0.227	1730.0
**Mean:**	**250.3**		**0.232**	
**Std. Dev.:**	**0.1**		**0.006**	
19	300.3	Cowan + pc.	0.202	1730.0
20	300.3	Cowan + pc.	0.195	1730.0
21	300.2	Cowan + pc.	0.197	1730.0
**Mean:**	**300.3**		**0.198**	
**Std. Dev.:**	**0.1**		**0.003**	
22	350.5	Cowan + pc.	0.176	1730.0
23	350.4	Cowan + pc.	0.173	1730.0
24	350.3	Cowan + pc.	0.173	1730.0
**Mean:**	**350.4**		**0.174**	
**Std. Dev.:**	**0.1**		**0.002**	
25	400.5	Cowan + pc.	0.159	1730.0
26	400.4	Cowan + pc.	0.157	1730.0
27	400.3	Cowan + pc.	0.155	1730.0
**Mean:**	**400.4**		**0.157**	
**Std. Dev.:**	**0.1**		**0.002**	
28	450.7	Cowan + pc.	0.148	1730.0
29	450.5	Cowan + pc.	0.141	1730.0
30	450.4	Cowan + pc.	0.143	1730.0
**Mean:**	**450.5**		**0.144**	
**Std. Dev.:**	**0.2**		**0.004**	
31	500.6	Cowan + pc.	0.142	1730.0

Created with NETZSCH Proteus software

Shot number	Temperature °C	Model	Diffusivity mm^2/s	Laser voltage V
32	500.5	Cowan + pc.	0.146	1730.0
33	500.4	Cowan + pc.	0.146	1730.0
**Mean:**	**500.5**		**0.145**	
**Std. Dev.:**	**0.1**		**0.002**	
34	510.3	Cowan + pc.	0.142	1730.0
35	510.2	Cowan + pc.	0.143	1730.0
36	510.2	Cowan + pc.	0.141	1730.0
**Mean:**	**510.3**		**0.142**	
**Std. Dev.:**	**0.1**		**0.001**	
37	520.3	Cowan + pc.	0.135	1730.0
38	520.2	Cowan + pc.	0.134	1730.0
39	520.1	Cowan + pc.	0.134	1730.0
**Mean:**	**520.2**		**0.134**	
**Std. Dev.:**	**0.1**		**0.001**	
40	530.3	Cowan + pc.	0.169	1730.0
41	530.2	Cowan + pc.	0.174	1730.0
42	530.2	Cowan + pc.	0.176	1730.0
**Mean:**	**530.2**		**0.173**	
**Std. Dev.:**	**0.1**		**0.004**	
43	540.3	Cowan + pc.	0.187	1730.0
44	540.2	Cowan + pc.	0.188	1730.0
45	540.2	Cowan + pc.	0.188	1730.0
**Mean:**	**540.3**		**0.188**	
**Std. Dev.:**	**0.1**		**0.001**	

Created with NETZSCH Proteus software

Thermal Diffusivity - NETZSCH LFA Analysis

General information

**Database :**	yklee.mdb	**Operator :**	YKLee
**Instrument :**	LFA 457	**Customer :**	YKLee
**Identity :**	SNU SnSe	**Remark(mment) :**	w/ graphite
**Date :**	12/8/2019	**Cp table :**	Cp_const
**Material :**	purified Na0.03Sn0.965Se	**Expansion table :**	undefined
**Ref. density (20.0 °C) /(g/cm^3) :**	5.910	**Temp. recalib. file :**	undefined
**Sample :**	purified Na0.03Sn0.965Se	**Purge gas :**	used
**Type :**	Single layer	**Furnace :**	LFA 457 Medium Rg
**Coating :**	yes	**Sample holder :**	unknown
**Thickness (RT) /mm :**	1.3000	**Laser :**	LFA 457 Laser
**Diameter /mm :**	8.000	**Centering cone :**	Customer
**Sensor :**	InSb	**Center cone ratio :**	0.74
**Beam enlargement : /mm**	12.7	**Furnace TC :**	S
**Laser filter : /%**	100.0	**Sample TC :**	S
**Atmosphere :**	Ar	**Sample Xp / Tn :**	2.40 / 0.60
**Gas flow : /(ml/min)**	100.00	**Furnace Xp / Tn :**	3.00 / 0.60
**Laboratory :**	EML	**Calculation code :**	C+p/l/0-x-0

Results

Shot number	Temperature °C	Model	Diffusivity mm^2/s	Laser voltage V
1	32.5	Cowan + pc.	0.450	1730.0
2	32.3	Cowan + pc.	0.435	1730.0
3	32.1	Cowan + pc.	0.436	1730.0
**Mean:**	**32.3**		**0.440**	
**Std. Dev.:**	**0.2**		**0.009**	
4	51.4	Cowan + pc.	0.415	1730.0
5	50.7	Cowan + pc.	0.424	1730.0
6	50.2	Cowan + pc.	0.415	1730.0
**Mean:**	**50.8**		**0.418**	
**Std. Dev.:**	**0.6**		**0.005**	
7	102.5	Cowan + pc.	0.359	1730.0
8	100.4	Cowan + pc.	0.361	1730.0
9	100.3	Cowan + pc.	0.364	1730.0
**Mean:**	**101.1**		**0.362**	
**Std. Dev.:**	**1.2**		**0.003**	
10	150.4	Cowan + pc.	0.321	1730.0
11	150.1	Cowan + pc.	0.323	1730.0
12	150.1	Cowan + pc.	0.322	1730.0
**Mean:**	**150.2**		**0.322**	
**Std. Dev.:**	**0.1**		**0.001**	
13	200.3	Cowan + pc.	0.286	1730.0
14	200.2	Cowan + pc.	0.281	1730.0
15	200.1	Cowan + pc.	0.283	1730.0
**Mean:**	**200.2**		**0.283**	
**Std. Dev.:**	**0.1**		**0.002**	
16	250.3	Cowan + pc.	0.246	1730.0
17	250.2	Cowan + pc.	0.239	1730.0
18	250.2	Cowan + pc.	0.240	1730.0
**Mean:**	**250.2**		**0.241**	
**Std. Dev.:**	**0.1**		**0.004**	
19	300.6	Cowan + pc.	0.212	1730.0
20	300.4	Cowan + pc.	0.207	1730.0
21	300.3	Cowan + pc.	0.207	1730.0
**Mean:**	**300.5**		**0.209**	
**Std. Dev.:**	**0.2**		**0.003**	
22	350.7	Cowan + pc.	0.184	1730.0
23	350.5	Cowan + pc.	0.183	1730.0
24	350.4	Cowan + pc.	0.181	1730.0
**Mean:**	**350.5**		**0.183**	
**Std. Dev.:**	**0.2**		**0.002**	
25	400.9	Cowan + pc.	0.169	1730.0
26	400.6	Cowan + pc.	0.165	1730.0
27	400.5	Cowan + pc.	0.163	1730.0
**Mean:**	**400.7**		**0.165**	
**Std. Dev.:**	**0.2**		**0.003**	
28	450.9	Cowan + pc.	0.155	1730.0
29	450.6	Cowan + pc.	0.152	1730.0
30	450.5	Cowan + pc.	0.152	1730.0
**Mean:**	**450.7**		**0.153**	
**Std. Dev.:**	**0.2**		**0.002**	
31	501.0	Cowan + pc.	0.148	1730.0

Created with NETZSCH Proteus software

Shot number	Temperature °C	Model	Diffusivity mm^2/s	Laser voltage V
32	500.7	Cowan + pc.	0.146	1730.0
33	500.5	Cowan + pc.	0.145	1730.0
**Mean:**	**500.8**		**0.146**	
**Std. Dev.:**	**0.3**		**0.002**	
34	511.2	Cowan + pc.	0.144	1730.0
35	510.8	Cowan + pc.	0.144	1730.0
36	510.6	Cowan + pc.	0.144	1730.0
**Mean:**	**510.8**		**0.144**	
**Std. Dev.:**	**0.3**		**0.000**	
37	521.2	Cowan + pc.	0.139	1730.0
**Mean:**	**521.2**		**0.139**	
**Std. Dev.:**	**0.0**		**0.000**	
38	531.2	Cowan + pc.	0.135	1730.0
39	530.7	Cowan + pc.	0.133	1730.0
40	530.5	Cowan + pc.	0.133	1730.0
**Mean:**	**530.8**		**0.133**	
**Std. Dev.:**	**0.3**		**0.001**	

Created with NETZSCH Proteus software

Thermal Diffusivity - NETZSCH LFA Analysis

General information

**Database :**	yklee.mdb	**Operator :**	YKLee
**Instrument :**	LFA 457	**Customer :**	YKLee
**Identity :**	SNU SnSe	**Remark(mment) :**	w/ graphite
**Date :**	12/20/2019	**Cp table :**	Cp_const
**Material :**	purified Na0.03Sn0.965Se	**Expansion table :**	undefined
**Ref. density (20.0 °C) /(g/cm^3) :**	5.910	**Temp. recalib. file :**	undefined
**Sample :**	purified Na0.03Sn0.965Se	**Purge gas :**	used
**Type :**	Single layer	**Furnace :**	LFA 457 Medium Rg
**Coating :**	yes	**Sample holder :**	unknown
**Thickness (RT) /mm :**	2.0100	**Laser :**	LFA 457 Laser
**Diameter /mm :**	8.000	**Centering cone :**	Customer
**Sensor :**	InSb	**Center cone ratio :**	0.74
**Beam enlargement : /mm**	12.7	**Furnace TC :**	S
**Laser filter : /%**	100.0	**Sample TC :**	S
**Atmosphere :**	Ar	**Sample Xp / Tn :**	2.40 / 0.60
**Gas flow : /(ml/min)**	100.00	**Furnace Xp / Tn :**	3.00 / 0.60
**Laboratory :**	EML	**Calculation code :**	C+p/mx/0-x-0

Results

Shot number	Temperature °C	Model	Diffusivity mm^2/s	Laser voltage V
1	32.2	Cowan + pc.	0.484	1730.0
2	33.1	Cowan + pc.	0.412	1730.0
3	33.3	Cowan + pc.	0.429	1730.0
**Mean:**	**32.8**		**0.441**	
**Std. Dev.:**	**0.6**		**0.038**	
4	52.1	Cowan + pc.	0.440	1730.0
5	51.7	Cowan + pc.	0.434	1730.0
6	50.8	Cowan + pc.	0.420	1730.0
**Mean:**	**51.5**		**0.431**	
**Std. Dev.:**	**0.7**		**0.010**	
7	101.0	Cowan + pc.	0.367	1730.0
8	100.9	Cowan + pc.	0.364	1730.0
9	100.8	Cowan + pc.	0.356	1730.0
**Mean:**	**100.9**		**0.362**	
**Std. Dev.:**	**0.1**		**0.006**	
10	151.2	Cowan + pc.	0.314	1730.0
11	151.0	Cowan + pc.	0.307	1730.0
12	150.7	Cowan + pc.	0.304	1730.0
**Mean:**	**151.0**		**0.309**	
**Std. Dev.:**	**0.2**		**0.005**	
13	201.4	Cowan + pc.	0.265	1730.0
14	201.1	Cowan + pc.	0.272	1730.0
15	200.9	Cowan + pc.	0.263	1730.0
**Mean:**	**201.1**		**0.267**	
**Std. Dev.:**	**0.2**		**0.005**	
16	251.3	Cowan + pc.	0.227	1730.0
17	251.0	Cowan + pc.	0.222	1730.0
18	250.7	Cowan + pc.	0.220	1730.0
**Mean:**	**251.0**		**0.223**	
**Std. Dev.:**	**0.3**		**0.004**	
19	301.1	Cowan + pc.	0.204	1730.0
20	300.8	Cowan + pc.	0.193	1730.0
21	300.6	Cowan + pc.	0.197	1730.0
**Mean:**	**300.8**		**0.198**	
**Std. Dev.:**	**0.3**		**0.005**	
22	351.2	Cowan + pc.	0.187	1730.0
23	351.0	Cowan + pc.	0.175	1730.0
24	350.7	Cowan + pc.	0.168	1730.0
**Mean:**	**351.0**		**0.177**	
**Std. Dev.:**	**0.2**		**0.009**	
25	401.2	Cowan + pc.	0.186	1730.0
26	401.1	Cowan + pc.	0.164	1730.0
27	400.8	Cowan + pc.	0.155	1730.0
**Mean:**	**401.0**		**0.168**	
**Std. Dev.:**	**0.2**		**0.016**	
28	450.9	Cowan + pc.	0.149	1730.0
29	450.7	Cowan + pc.	0.147	1730.0
30	450.5	Cowan + pc.	0.149	1730.0
**Mean:**	**450.7**		**0.148**	
**Std. Dev.:**	**0.2**		**0.001**	
31	501.0	Cowan + pc.	0.134	1730.0

Created with NETZSCH Proteus software

Shot number	Temperature °C	Model	Diffusivity mm^2/s	Laser voltage V
32	500.9	Cowan + pc.	0.146	1730.0
33	500.7	Cowan + pc.	0.139	1730.0
**Mean:**	**500.9**		**0.140**	
**Std. Dev.:**	**0.2**		**0.006**	
34	510.0	Cowan + pc.	0.150	1730.0
35	510.2	Cowan + pc.	0.148	1730.0
36	510.2	Cowan + pc.	0.141	1730.0
**Mean:**	**510.1**		**0.147**	
**Std. Dev.:**	**0.1**		**0.004**	
37	520.1	Cowan + pc.	0.126	1730.0
38	520.1	Cowan + pc.	0.123	1730.0
39	520.1	Cowan + pc.	0.122	1730.0
**Mean:**	**520.1**		**0.124**	
**Std. Dev.:**	**0.0**		**0.002**	
40	530.1	Cowan + pc.	0.172	1730.0
41	530.1	Cowan + pc.	0.184	1730.0
42	530.1	Cowan + pc.	0.187	1730.0
**Mean:**	**530.1**		**0.181**	
**Std. Dev.:**	**0.0**		**0.008**	
43	540.1	Cowan + pc.	0.150	1730.0
44	540.1	Cowan + pc.	0.196	1730.0
45	540.1	Cowan + pc.	0.194	1730.0
**Mean:**	**540.1**		**0.180**	
**Std. Dev.:**	**0.0**		**0.026**	

Created with NETZSCH Proteus software

Thermal Diffusivity - NETZSCH LFA Analysis

General information

**Database :**	yklee.mdb	**Operator :**	YKLee
**Instrument :**	LFA 457	**Customer :**	YKLee
**Identity :**	SNU SnSe	**Remark(mment) :**	w/ graphite
**Date :**	12/12/2019	**Cp table :**	Cp_const
**Material :**	purified Na0.03Sn0.965Se	**Expansion table :**	undefined
**Ref. density (20.0 °C) /(g/cm^3) :**	5.910	**Temp. recalib. file :**	undefined
**Sample :**	purified Na0.03Sn0.965Se	**Purge gas :**	used
**Type :**	Single layer	**Furnace :**	LFA 457 Medium Rg
**Coating :**	yes	**Sample holder :**	unknown
**Thickness (RT) /mm :**	1.7400	**Laser :**	LFA 457 Laser
**Diameter /mm :**	8.000	**Centering cone :**	Customer
**Sensor :**	InSb	**Center cone ratio :**	0.74
**Beam enlargement : /mm**	12.7	**Furnace TC :**	S
**Laser filter : /%**	100.0	**Sample TC :**	S
**Atmosphere :**	Ar	**Sample Xp / Tn :**	2.40 / 0.60
**Gas flow : /(ml/min)**	100.00	**Furnace Xp / Tn :**	3.00 / 0.60
**Laboratory :**	EML	**Calculation code :**	C+p/mx/0-x-0

Results

**Shot number**	**Temperature** °C	**Model**	**Diffusivity** mm^2/s	**Laser voltage** V
1	37.8	Cowan + pc.	0.465	1730.0
2	37.4	Cowan + pc.	0.469	1730.0
3	36.5	Cowan + pc.	0.486	1730.0
**Mean:**	**37.3**		**0.473**	
**Std. Dev.:**	**0.6**		**0.011**	
4	51.6	Cowan + pc.	0.475	1730.0
5	51.1	Cowan + pc.	0.459	1730.0
6	50.2	Cowan + pc.	0.478	1730.0
**Mean:**	**51.0**		**0.471**	
**Std. Dev.:**	**0.7**		**0.010**	
7	100.8	Cowan + pc.	0.404	1730.0
8	100.7	Cowan + pc.	0.393	1730.0
9	100.6	Cowan + pc.	0.386	1730.0
**Mean:**	**100.7**		**0.394**	
**Std. Dev.:**	**0.1**		**0.009**	
10	151.0	Cowan + pc.	0.342	1730.0
11	150.9	Cowan + pc.	0.338	1730.0
12	150.8	Cowan + pc.	0.341	1730.0
**Mean:**	**150.9**		**0.340**	
**Std. Dev.:**	**0.1**		**0.002**	
13	201.3	Cowan + pc.	0.295	1730.0
14	201.1	Cowan + pc.	0.293	1730.0
15	200.8	Cowan + pc.	0.287	1730.0
**Mean:**	**201.1**		**0.292**	
**Std. Dev.:**	**0.2**		**0.004**	
16	251.4	Cowan + pc.	0.242	1730.0
17	251.1	Cowan + pc.	0.238	1730.0
18	250.9	Cowan + pc.	0.237	1730.0
**Mean:**	**251.1**		**0.239**	
**Std. Dev.:**	**0.3**		**0.002**	
19	301.3	Cowan + pc.	0.202	1730.0
20	301.0	Cowan + pc.	0.197	1730.0
21	300.8	Cowan + pc.	0.189	1730.0
**Mean:**	**301.0**		**0.196**	
**Std. Dev.:**	**0.3**		**0.007**	
22	351.4	Cowan + pc.	0.187	1730.0
23	351.1	Cowan + pc.	0.176	1730.0
24	350.9	Cowan + pc.	0.170	1730.0
**Mean:**	**351.1**		**0.178**	
**Std. Dev.:**	**0.3**		**0.009**	
25	401.2	Cowan + pc.	0.168	1730.0
26	401.0	Cowan + pc.	0.162	1730.0
27	400.7	Cowan + pc.	0.162	1730.0
**Mean:**	**401.0**		**0.164**	
**Std. Dev.:**	**0.3**		**0.004**	
28	451.1	Cowan + pc.	0.162	1730.0
29	450.9	Cowan + pc.	0.152	1730.0
30	450.7	Cowan + pc.	0.154	1730.0
**Mean:**	**450.9**		**0.156**	
**Std. Dev.:**	**0.2**		**0.005**	
31	501.0	Cowan + pc.	0.158	1730.0

Created with NETZSCH Proteus software

Shot number	Temperature °C	Model	Diffusivity mm^2/s	Laser voltage V
32	500.8	Cowan + pc.	0.149	1730.0
33	500.6	Cowan + pc.	0.144	1730.0
**Mean:**	**500.8**		**0.150**	
**Std. Dev.:**	**0.2**		**0.007**	
34	510.4	Cowan + pc.	0.153	1730.0
35	510.3	Cowan + pc.	0.150	1730.0
36	510.2	Cowan + pc.	0.144	1730.0
**Mean:**	**510.3**		**0.149**	
**Std. Dev.:**	**0.1**		**0.004**	
37	520.3	Cowan + pc.	0.144	1730.0
38	520.2	Cowan + pc.	0.136	1730.0
39	520.2	Cowan + pc.	0.137	1730.0
**Mean:**	**520.2**		**0.139**	
**Std. Dev.:**	**0.1**		**0.004**	
40	530.3	Cowan + pc.	0.177	1730.0
41	530.3	Cowan + pc.	0.173	1730.0
**Mean:**	**530.3**		**0.175**	
**Std. Dev.:**	**0.0**		**0.003**	

Created with NETZSCH Proteus software

Supplementary Table 1. The sample densities for purified SnSe and $Na_xSn_{0.995-x}Se$ samples (x = 0.01, 0.02, 0.03). Their relative densities are around 96%. The theoretical density of single-crystal SnSe is 6.18 g·cm^{-3}.

Sample	Density (g·cm^{-3})	Relative density (%)
SnSe	5.921	95.8
$Na_{0.01}Sn_{0.985}Se$	5.942	96.1
$Na_{0.02}Sn_{0.975}Se$	5.921	95.8
$Na_{0.03}Sn_{0.965}Se$	5.914	95.6

References

1. Blöchl, P. E. Projector Augmented-Wave Method. *Phys. Rev. B* **50**, 17953–17979 (1994).
2. Peng, H., Yang, Z. H., Perdew, J. P. & Sun, J. Versatile van Der Waals Density Functional Based on a Meta-Generalized Gradient Approximation. *Phys. Rev. X* **6**, 041005 (2016).
3. Kresse, G. & Hafner, J. Norm-Conserving and Ultrasoft Pseudopotentials for First-Row and Transition Elements. *J. Phys. Condens. Matter* **6**, 8245–8257 (1994).
4. Kresse, G. & Furthmüller, J. Efficient Iterative Schemes for Ab Initio Total-Energy Calculations Using a Plane-Wave Basis Set. *Phys. Rev. B* **54**, 11169–11186 (1996).
5. Lany, S. & Zunger, A. Assessment of Correction Methods for the Band-Gap Problem and for Finite-Size Effects in Supercell Defect Calculations: Case Studies for ZnO and GaAs. *Phys. Rev. B - Condens. Matter Mater. Phys.* **78**, 235104 (2008).
6. Roy, S., Joshi, A. G., Chatterjee, S. & Ghosh, A. K. Local Symmetry Breaking in SnO_2 Nanocrystals with Cobalt Doping and Its Effect on Optical Properties. *Nanoscale* **10**, 10664–10682 (2018).
7. Ferreira, C. S., Santos, P. L., Bonacin, J. A., Passos, R. R. & Pocrifka, L. A. Rice Husk Reuse in the Preparation of SnO_2/SiO_2 Nanocomposite. *Mater. Res.* **18**, 639–643 (2015).
8. Rao, G. T., Babu, B., Ravikumar, R. V. S. S. N., Shim, J. & Reddy, C. V. Structural and Optical Properties of Fe-Doped SnO_2 Quantum Dots. *Mater. Res. Express* **4**, 125021 (2017).
9. Yang, S. & Gao, L. Facile and Surfactant-Free Route to Nanocrystalline Mesoporous Tin Oxide. *J. Am. Ceram. Soc.* **89**, 1742–1744 (2006).
10. Zhao, L.-D. *et al.* Ultralow Thermal Conductivity and High Thermoelectric Figure of Merit in SnSe Crystals. *Nature* **508**, 373–377 (2014).
11. Zhao, L.-D. *et al.* Ultrahigh Power Factor and Thermoelectric Performance in Hole-Doped Single-Crystal SnSe. *Science* **351**, 141–144 (2016).
12. Lee, Y. K., Luo, Z., Cho, S. P., Kanatzidis, M. G. & Chung, I. Surface Oxide Removal for Polycrystalline SnSe Reveals Near-Single-Crystal Thermoelectric Performance. *Joule* **3**, 719–731 (2019).
13. Tan, G. *et al.* Non-Equilibrium Processing Leads to Record High Thermoelectric Figure of Merit in PbTe–SrTe. *Nat. Commun.* **7**, 12167 (2016).
14. Olvera, A. A. *et al.* Partial Indium Solubility Induces Chemical Stability and Colossal Thermoelectric Figure of Merit in Cu_2Se. *Energy Environ. Sci.* **10**, 1668–1676 (2017).
